# Structural insight into SUMO chain recognition and manipulation by the ubiquitin ligase RNF4

**DOI:** 10.1038/ncomms5217

**Published:** 2014-06-27

**Authors:** Yingqi Xu, Anna Plechanovová, Peter Simpson, Jan Marchant, Orsolya Leidecker, Sebastian Kraatz, Ronald T. Hay, Steve J. Matthews

**Affiliations:** 1 Centre for Structural Biology, Department of Life Sciences, Imperial College London, South Kensington, London SW7 2AZ, UK; 2 Centre for Gene Regulation and Expression, College of Life Sciences, University of Dundee, Dow Street, Dundee DD1 5EH, UK; 3 These authors contributed equally to this work

## Abstract

The small ubiquitin-like modifier (SUMO) can form polymeric chains that are important signals in cellular processes such as meiosis, genome maintenance and stress response. The SUMO-targeted ubiquitin ligase RNF4 engages with SUMO chains on linked substrates and catalyses their ubiquitination, which targets substrates for proteasomal degradation. Here we use a segmental labelling approach combined with solution nuclear magnetic resonance (NMR) spectroscopy and biochemical characterization to reveal how RNF4 manipulates the conformation of the SUMO chain, thereby facilitating optimal delivery of the distal SUMO domain for ubiquitin transfer.

## Abstract (web summary)

SUMO forms flexible polymeric chains that can interact with ubiquitin ligases, such as RNF4. Here Xu *et al*. have used NMR spectroscopy and biochemical experiments to investigate the interaction between SUMO and RNF4, and propose a mechanism for delivery of substrates to the ubiquitination machinery.

## Graphical abstract

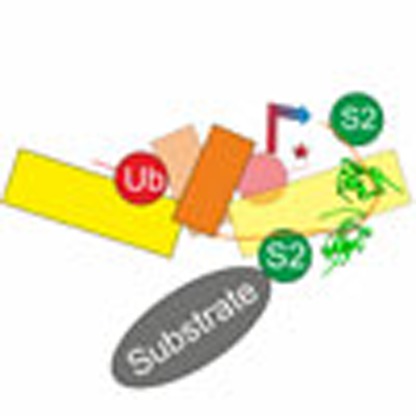

Conjugation of ubiquitin(s) to proteins (ubiquitination) provides a fast and reversible mechanism to regulate the stability, location and activity of substrate proteins^1^. Best known for its role in protein degradation^2^, ubiquitination is also involved in many other cellular activities, such as DNA repair, transcriptional regulation, signal transduction, apoptosis, endocytosis and immune response^3^. Other ubiquitin-like proteins can also be covalently attached to a range of target proteins^4^. For example, small ubiquitin-like modifier (SUMO)^5^ has been found to modify over 1,000 substrates, and a dedicated three-step cascade involving an activating enzyme (E1), a conjugating enzyme (E2) and a protein ligase (E3) similar to the ubiquitin conjugation pathway is responsible for SUMOylation^6^,^7^. Similar to ubiquitination, SUMOylation involves the formation of an isopeptide bond between the C-terminal glycine and the side chain of a lysine residue from the substrate protein. In most cases, this lysine resides within a sequence with consensus ψ-K-x-D/E, where ψ is a hydrophobic residue^8^. In contrast with ubiquitin chains, where linkages through all seven lysine residues have been observed^9^, one type of SUMO chain seems to predominate, where linkage is through lysine 11 of SUMO-2/3 (refs 10, 11).

The signalling of downstream functions by ubiquitination and SUMOylation relies on the ability of modified substrates to interact with appropriate effector proteins. The various types of ubiquitin linkages adopt different overall shapes^12^,^13^ that are usually read out by specific arrangements of ubiquitin-binding motifs^14^ and lead to very different signalling outputs^15^,^16^. SUMO has also been found to be rather versatile and regulates transcription, DNA repair, chromosome segregation, as well as the sorting of proteins to distinct cellular destinations^6^. Although many of these functions involve monomeric SUMOylation, the roles of SUMO chains are becoming equally important and increasingly understood^17^. For example, the polySUMOylated promyelocytic leukaemia protein (PML) is preferentially recognized by the RING Finger Protein 4 (RNF4), a ubiquitin ligase (E3), and is subsequently degraded by the ubiquitin–proteasome pathway^18^. This process is critical for successful treatment of acute promyelocytic leukaemia with arsenic. In fact, RNF4 belongs to a family of proteins called SUMO-targeted ubiquitin ligases, which also comprises yeast proteins such as Slx5, Slx8, Rfp1 and Rfp2 (refs 19, 20, 21). These proteins have been shown to help maintain genomic stability in yeast, and their deletion results in the accumulation of high-molecular weight polySUMO conjugates. Although SUMO was initially suggested to protect some proteins from ubiquitination by shielding the conjugation sites, it is now clear that the SUMO chains can act as a signal for ubiquitination. SUMO chains also interact with other proteins such as Zip1 and Red1 from the synaptonemal complex^22^,^23^, the microtubule motor protein CENP-E^24^ and Uls1, which has both SUMO-targeted ubiquitin ligase and translocase activities^25^. Through these interactions, SUMO chains exert effects on meiosis and chromatin structure. Recently, additional polySUMO-binding proteins, including Arkadia, FLASH, C5orf25 and SOBP, have been identified through a computational string search^26^.

The SUMO Interaction Motif (SIM) is a sequence that binds specifically to SUMO domains^27^. A series of biochemical and structural studies^28^,^29^,^30^,^31^,^32^,^33^,^34^ have established that a stretch of hydrophobic residues (V/I/L) form the SIM core and bind into a groove between the second β-strand and the following α-helix of SUMO. Some SIMs feature a negatively charged tract flanking these hydrophobic residues that which enhances the binding through electrostatic interactions as well as dictating the orientation of binding. Depending on their particular sequences, SIMs bind to SUMOs with affinity constants in the milli- to micromolar range and can demonstrate preference to either SUMO-1 or SUMO-2/3. For proteins with multiple SIMs, such as RNF4, their binding to polySUMO is much stronger than to monoSUMO.

Although a crystal structure of dimeric RNF4–RING in complex with an ubiquitin-loaded E2 has provided us with a high-resolution snapshot of the priming mechanism for ubiquitin transfer^35^,^36^, the dynamic nature of the polySUMO-modified substrates has precluded crystallographic investigation into their delivery to this machinery. Work presented here provides new insight into SUMO chain recognition and how the SUMO chain may be delivered to this ubiquitin ligation machinery. Segmental labelling together with multidimensional NMR was used to study Lys11-linked SUMO-2 dimers and to investigate the structure of their complex with a peptide containing SIM2 and SIM3 (SIM2,3) from RNF4. Although the two SUMO subunits appear to move freely with respect to each other in solution, they are significantly restrained upon binding SIM2,3. Such manipulation of SUMO chain conformation facilitates efficient ubiquitination of the distal SUMO domain by the RING-activated ubiquitin-loaded E2.

## Results

### Mapping the interaction between RNF4 and SUMO-2 chains

**Figure 1:**
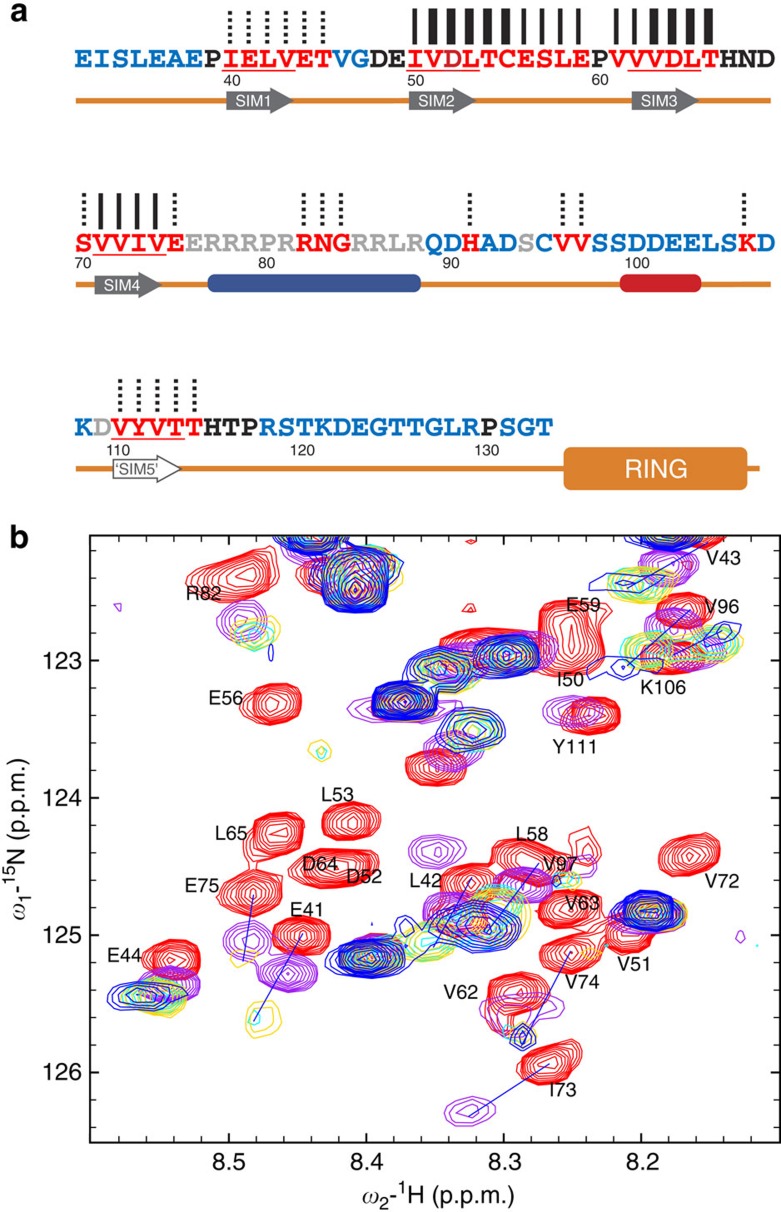
NMR mapping the interaction with SUMO-2 chains on the RNF4–SIM region. (**a**) Amino-acid sequence for the N-terminal fragment of RNF4 used in this study. The four SIMs as well as ‘SIM5’ are underlined; the residues affected by titration are coloured red, whereas those unaffected are coloured blue; residues coloured black are not classified owing to lack of assignment or overlap. A dashed line over the affected residue means its position was shifted with increasing SUMO concentrations; a thin line means the peak was initially shifted, then disappeared after ~1:1 SUMO added; a thick line indicates that the peak disappeared when <0.3 molar equivalents of SUMO was added. A schematic representation for RNF4 is shown below the sequence, where the SIMs are supposed to form β-strands upon binding to SUMO; a very positive region (with eight arginines) and a negative stretch (the two serine residues may be phosphorylated) are also highlighted in the scheme. (**b**) Overlay of ^1^H–^15^N HSQC NMR spectra for free RNF4 32–133 (red), RNF4 32–133 titrated with SUMO monomer (magenta), SUMO dimer (cyan), SUMO dimer with truncations (yellow) and polySUMO chain (blue); the titrations are represented by the mixtures at 1 M equivalent SUMO (in monomer concentration). Labels are placed close to the peaks in the free state. The full spectra are also available as Supplementary Fig. 2.

Mammalian RNF4 sequences contain four SIMs in the N-terminal region (Fig. 1a) and a C-terminal RING domain responsible for dimerization and ubiquitination activity. To examine the folded state of RNF4 in solution, we used NMR spectroscopy. Two-dimensional (2D) ^1^H–^15^N heteronuclear single quantum coherence (HSQC) NMR spectra of RNF4_ΔN31_, which retains SUMO-targeted ubiquitination activity, reveals the folded C-terminal RING finger domain together with N-terminal region containing the SIMs that display NMR spectra with poor chemical shift dispersion, suggesting that this region is disordered in solution (Supplementary Fig. 1). To assign the N-terminal SIM region of RNF4 and monitor polySUMO binding, we focused our NMR analysis on the flexible N-terminal region alone (residues 32–133 of RNF4; Fig. 1a). Despite the poor dispersion of signals for RNF4_32—133_, ~90 individual peaks could be observed and assigned using a combination of standard triple resonance approach and HNN and HN(C)N experiments optimized for unfolded polypeptides^37^. The majority of backbone resonances for the N-terminal region of RNF4 were assigned (Supplementary Fig. 2), which allowed us to map the interaction of RNF4 with SUMO in detail.

2D ^1^H–^15^N HSQC NMR spectra were recorded of ^13^C,^15^N-labelled RNF4_32–133_ in the presence of increasing amounts of unlabelled SUMO-2 monomer, full-length SUMO-2 dimer and SUMO-2 dimer prepared using truncated SUMO-2s (SUMO-2_ΔN11_ and SUMO-2_ΔGG_; see Methods section), as well as SUMO-2 chains (Fig. 1b and Supplementary Fig. 2). Binding of SUMO-2 caused significant chemical shift perturbations in RNF4_32–133_, with many peaks moving or completely disappearing over the course of the titration. The same peaks are affected by titrations with any of all four different SUMO-2 forms; in particular, residues from all four SIMs showed binding (Fig. 1). The binding affinity, however, appears to be different among individual SIMs and for the different polymer states of SUMO-2. As can be seen in Fig. 1b (and Supplementary Fig. 2), peaks corresponding to SIM2 (I50, V51, D52, L53 and T54) and SIM3 (V62, V63, D64, L65 and T66) disappear completely after adding one molar equivalent of SUMO-2 (as monomer concentration). In fact, these peaks had disappeared much earlier in the titration, with SIM2 peaks broadened beyond detection at ~0.1 molar equivalent and SIM3 peaks at ~0.2. Peak positions for SIM4 (V71, V72, I73 and V74) and SIM1 (I40, E41, L42 and V43) are shifted and gradually weakened during the titration with monomer SUMO-2, suggesting that they have weaker binding affinity, which likely results from the significant deviation from optimal SIM sequences^26^,^32^. Although the measurement of accurate dissociation constants from the NMR data is not possible because of intermediate exchange regimes, the relative affinities are SIM2>SIM3>>SIM1/SIM4. Different affinities were also observed for the four different polymeric states of SUMO-2, with Lys11-linked tetra-SUMO greater than diSUMO and monoSUMO being the weakest. To quantify the relative affinities of mono-, di- and tetraSUMO-2 for RNF4_32–133_, we used isothermal calorimetry (Supplementary Fig. 3). Isopeptide-linked tetraSUMO-2 had an apparent *K*_D_s of 2.5 μM, whereas the apparent *K*_D_ for isopeptide-linked diSUMO-2 was 13.5 μM and for monoSUMO-2 59.5 μM. The NMR and isothermal calorimetry data highlight the role of multivalency in polySUMO recognition, and our values measured on isopeptide-linked chains are in line with those determined for the interaction of SIM2,3 with head-to-tail linear fusions of SUMO^38^. In Fig. 1b, some peaks (such as I73 and E41) disappear only when SUMO-2 dimers or polySUMO-2 chains were added. Despite the affinity differences, the perturbation patterns are very similar, which is most evident for those peaks (E41, V43 and so on in Fig. 1b), where the shifted peaks from different titrations appear along a common trajectory. Such a similarity implies that RNF4 recognizes the same sites on SUMO-2 whether it is present as a monomer or as part of a chain.

In addition to residues from the typical SIMs, several other residues are also perturbed in the titration (Fig. 1). In particular, all peaks from the linker region between SIM2 and SIM3 (C55, E56, S57, L58 and E59) are significantly affected, suggesting that these residues are in an altered conformation in the bound state. Perturbations were also observed for a contiguous hydrophobic region (V110, Y111 and V112) located in the long linker region connecting SIM4 and the RING domain. The VYV motif does not conform to the consensus sequence for SIMs, but could represent an additional binding site for SUMO. Perturbations are also observed within the arginine-rich region following SIM4, most notably residues R82, N83 and G84, which could indicate that this region is involved in binding SUMO-2 chains or undergoes a structural transition upon SUMO binding.

### SIM2 and SIM3 have a major role in binding to SUMO-2 chains

**Figure 2:**
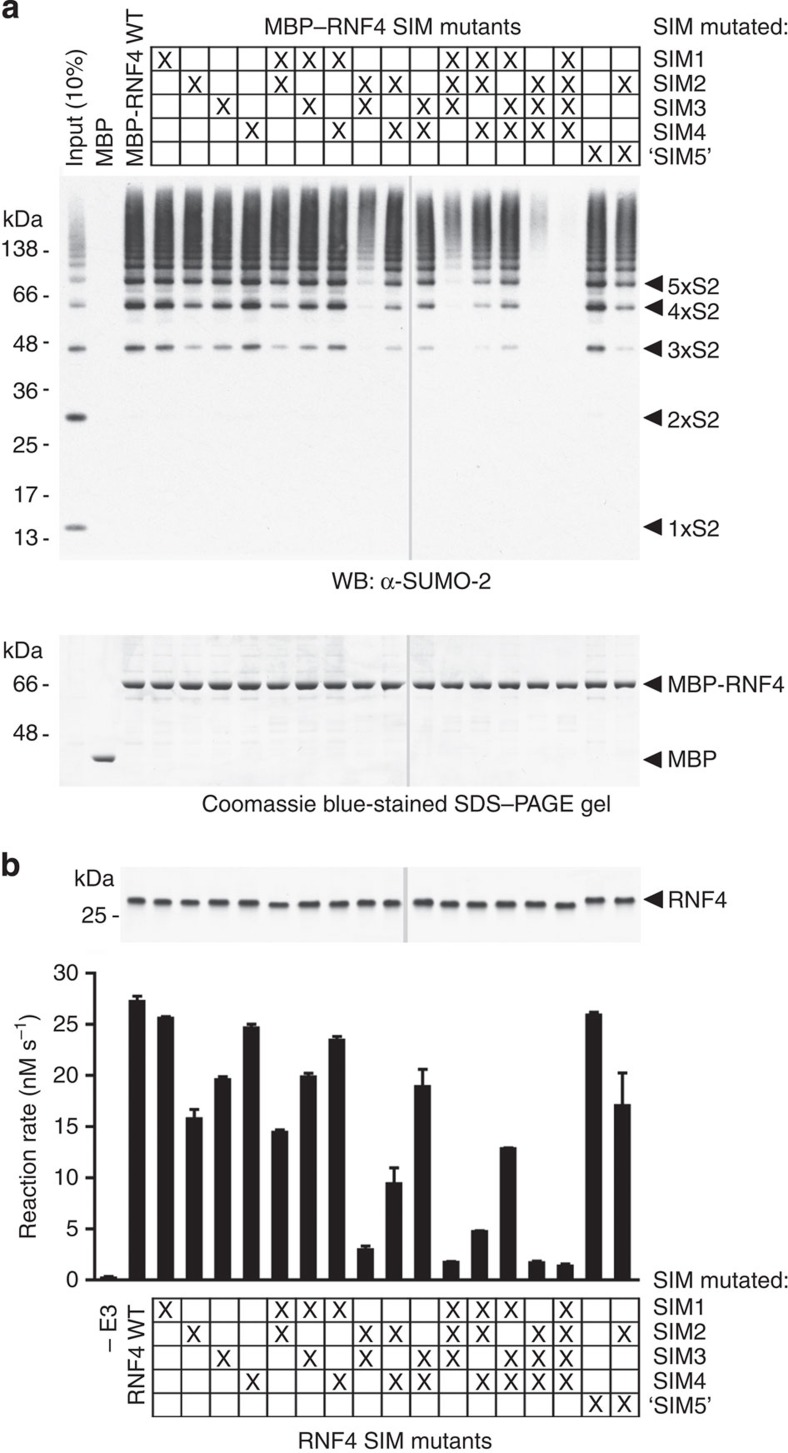
SIM2 and SIM3 are the key determinants for SUMO-2 chain binding and ubiquitination. (**a**) Pull-down experiment with MBP-tagged SIM mutants of RNF4. Bound material was analysed by SDS–PAGE and Coomassie blue staining (lower panel) or by immunostaining with anti-SUMO-2 antibody (upper panel). (**b**) Substrate ubiquitination activity of SIM mutants of RNF4. Data represent the mean of duplicate reactions with errors displayed as±s.d. Coomassie blue-stained SDS–PAGE gel with wild-type and mutant RNF4 proteins is shown as a loading control. Mutated SIMs are marked by X.

To further investigate the role of individual SIMs in RNF4 recognition of SUMO chains, a site-directed mutational analysis was performed. Various RNF4 SIM mutants were created by replacing central hydrophobic SIM residues with alanine and expressed as fusion proteins with maltose-binding protein (MBP), and assessed for binding to SUMO chains using pull-down assays. Although none of the individual SIMs from RNF4 is essential for the interaction with SUMO-2 chains (Fig. 2a), SIM2 and SIM3 appear to have the prominent role as mutation of either caused a significant decrease in binding to SUMO-2 trimers and tetramers, but the interaction with longer chains (>4) was not significantly affected. Mutation of the SIM2,3 pair abolished binding to trimeric and tetrameric SUMO-2, and reduced the interaction to longer chains, whereas RNF4 possessing only SIM2 or SIM3 was able to pull-down all SUMO-2 chains. Absence of either SIM1 or SIM4, or the SIM1,4 pair did not significantly affect the interaction with SUMO-2 chains in our assays. Mutation of the putative ‘SIM5’ did not have a detectable effect on the interaction with polySUMO-2 and is unlikely to be a bona fide functional SIM. Likewise mutation of the basic region distal to SIM4 had no impact on SUMO chain binding. In summary, the pull-down data imply the strength of binding between individual SIMs of RNF4 and SUMO-2 following the order: SIM2>SIM3>> SIM4>SIM1.

### Effects of SIM mutants on ubiquitination activities of RNF4

To assess the role of each SIM in substrate-targeted E3 ligase activity of RNF4, a series SIM mutants of RNF4 were created by mutating the central hydrophobic residues to alanine (Fig. 1) and these were tested in a single-turnover ubiquitination assay^35^ (Fig. 2b). Consistent with the pull-down experiments, a loss of any of the four canonical SIMs results in only a moderate decrease in substrate ubiquitination activity, and SIM2 appears to be the most important for substrate ubiquitination. RNF4 mutants possessing only SIM2 or SIM3 retained 50% and 17% of activity of the wild-type protein, respectively. Although SIM1 or SIM4 failed to promote ubiquitination in isolation, they contributed to substrate ubiquitination activity when either SIM2 or SIM3 was mutated. For example, an RNF4–SIM1,2 mutant showed ~50% of wild-type activity, but this dropped to 20% when combined with the mutation in SIM4. Taken together, the SUMO-targeted ubiquitination activity correlated well with binding to SUMO-2 chains. SIM2 and SIM3 are essential for efficient ubiquitination of the substrate, whereas SIM1 and SIM4 have a minor role. To confirm that the effects of these mutations were the result of SUMO-2 chain recruitment and not affecting the intrinsic ubiquitin E3 ligase activity of RNF4, an autoubiquitination assay was carried out^35^. As expected, all the SIM mutants of RNF4 showed autoubiquitination activity comparable to wild type (Supplementary Fig. 4).

### SUMO subunits are unrestrained in SUMO-2 dimers

**Figure 3:**
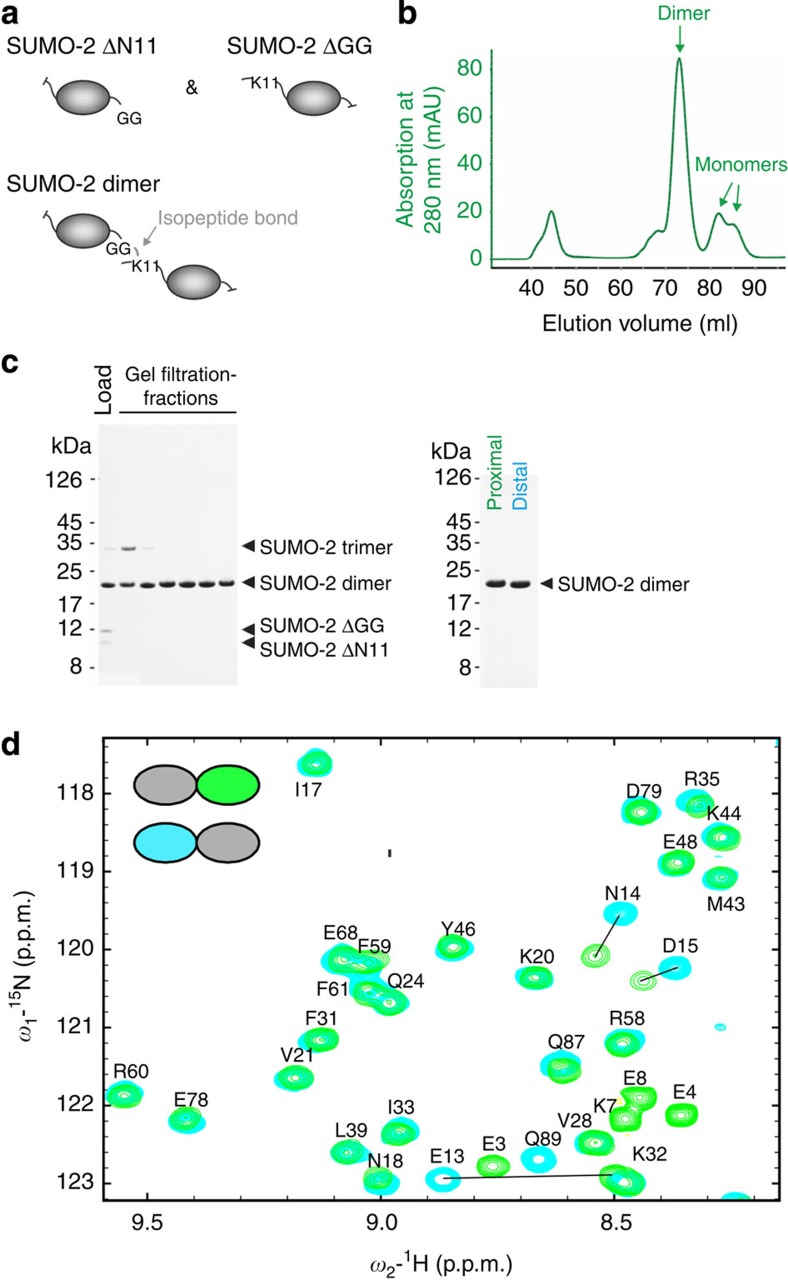
Preparation of segmentally labelled lys11-linked SUMO-2 dimers for NMR. (**a**) To increase efficiency of formation of SUMO-2 dimer, two truncated SUMO-2 constructs, termed SUMO-2 ΔN11 and SUMO-2 ΔGG, were designed. SUMO-2 ΔN11 lacks Lys11, and thus cannot be modified by another SUMO-2 molecule. SUMO-2 ΔGG has the C-terminal diglycine motif deleted and cannot be conjugated to target proteins. Together, these two constructs can form SUMO-2 dimers, but not longer chains. (**b**) Chromatogram showing purification of SUMO-2 dimer by gel filtration chromatography on a Superdex 75 column. (**c**) Coomassie blue-stained SDS–PAGE gel showing fractions from gel filtration chromatography (left) and purified SUMO-2 dimers for NMR studies (right). Lane 1: SUMO-2 dimer comprises ^13^C,^15^N-labelled proximal SUMO-2 ΔGG and unlabelled SUMO-2 ΔN11; lane 2: SUMO-2 dimer containing ^13^C,^15^N-labelled distal SUMO-2 ΔN11 and unlabelled SUMO-2 ΔGG. (**d**) Overlay of ^1^H–^15^N HSQC NMR spectra for the two SUMO-2 domains (distal SUMO-2 ΔN11 in cyan and proximal SUMO-2 ΔGG in green) in the K11-linked segmentally labelled SUMO-2 dimer. All peaks corresponding to the core residues 17–87 possess nearly identical chemical shifts in either SUMO-2 domain. Peaks that show significant difference correspond to residues either only present in one subunit owing to detection at the termini or very close to the isopeptide bond. These data suggest that the core SUMO domains are identical in structure and behave independently from each other.

To address whether polySUMO-2 chains adopt a specific quaternary structure that might be important for their recruitment to the RNF4 ubiquitin E3 ligase domain, we used NMR to determine the three-dimensional solution structure of a SUMO-2 dimer using a segmental labelling approach (Fig. 3a). In this scheme, two truncated SUMO-2 constructs were designed, SUMO-2 ΔN11 and SUMO-2 ΔGG. SUMO-2 ΔN11 is the distal domain as it lacks Lys11 and thus cannot be modified with another SUMO-2 molecule, whereas in the proximal domain the C-terminal diglycine motif is deleted (SUMO-2 ΔGG) and therefore cannot be conjugated to target proteins. Together, these two constructs can only form SUMO-2 dimers, but not longer chains (Fig. 3b,c).

^1^H–^15^N HSQC NMR experiments were recorded on two distinct Lys11-linked SUMO-2 dimers, each with only one subunit segmentally ^15^N,^13^C-labelled, whereas the other subunit remains unlabelled (Fig. 3d). The NMR spectra for both SUMO domains are very similar, and the majority of peaks overlay well. Spectral differences are due to chemical influences arising from isopeptide formation. In addition, comparison of the chemical shifts against monomer SUMO-2 shows a high degree of similarity, suggesting that each of the two SUMO subunits in the Lys11-linked dimer retains a similar structure to the monomer and remain independent to each other.

**Figure 4:**
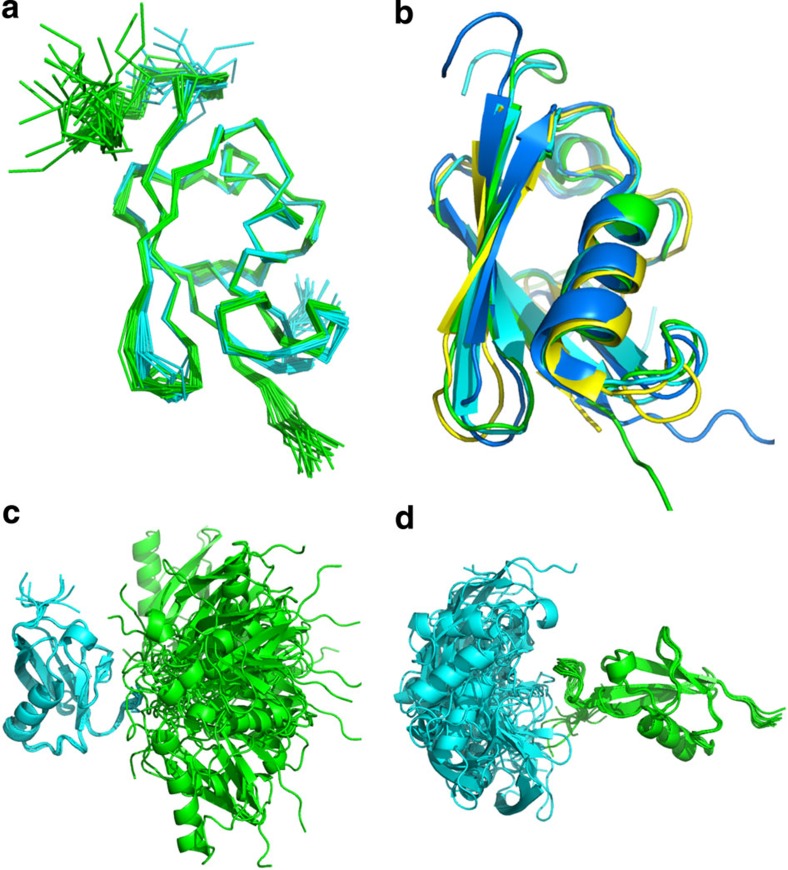
NMR solution structure of lys11-linked SUMO-2 dimer. (**a**) Superposition of the NMR ensembles for distal (SUMO-2 ΔN11, cyan) and proximal subunits (SUMO-2 ΔGG, green) highlights the structural similarity of the two domains. (**b**) Cartoon representation for the superposition of SUMO-2 dimer subunits (distal in cyan and proximal in green) and previously determined SUMO structures, namely SUMO-3 from 2RPQ (marine)^31^ and SUMO-2 from 1WM3 (yellow)^41^. The SUMO-2 domains within the SUMO-2 dimer are highly similar to reported structures for individual SUMO domains, as they superpose with an r.m.s.d. of 1.7 Å over 72 backbone atoms for SUMO-3 and 1.6 Å over 75 residues for SUMO-2. (**c**) The NMR ensemble of 20 structures for the individual SUMO domains within the SUMO-2 dimer superposed over equivalent atoms in the distal domains (SUMO-2 ΔN11, cyan) to illustrate the absence of a preferred relative orientation of the two SUMO-2 domains (**d**) The NMR ensemble for the SUMO-2 dimer superposed over equivalent atoms in the proximal domain (SUMO-2 ΔGG, green).

To characterize this further, the solution structure of the SUMO-2 dimer was determined using nuclear Overhauser effect (NOE) restraints automatically assigned by ARIA^39^ and backbone torsion angle restraints derived from chemical shifts^40^. The structures of both SUMO domains, as shown in Fig. 4a, are well defined, with root mean squared deviation (r.m.s.d.) for backbone atoms within secondary structures being 0.20 Å and 0.10 Å for the distal (SUMO-2 ΔN11, cyan) and proximal (SUMO-2 ΔGG, green) subunits, respectively. Apart from the N- and C termini, the two domains can be superimposed onto each other as well as previously determined SUMO structures (Fig. 4b), for SUMO-3 with the SIM from MCAF-1 bound (2RPQ)^31^, human SUMO-2 monomer (1WM3)^41^, and the free-linear diSUMO-2 (4BKG)^38^. The structures of each SUMO domains in Lys11-linked diSUMO-2 superpose with an r.m.s.d. of 1.7 Å over 72 backbone atoms on SUMO-3, 1.6 Å over 75 residues for the SUMO-2 monomer^31^,^41^ and 1.4 Å over 73 residues for the single domain in the asymmetric unit of the linear diSUMO-2 (ref. 38) crystal structure. The small deviations in these structures are likely a reflection of the precision of NMR structures rather than true differences. The relative domain orientation within the SUMO-2 dimer ensemble is not well defined (Fig. 4c,d). Rotational correlation times for the two subunits, derived from NMR relaxation data, are calculated as 7.5±0.2 and 9.0±0.1 ns for distal (SUMO-2 ΔN11) and proximal (SUMO-2 ΔGG) domains, respectively, (Supplementary Fig. 5). The long and flexible N-terminal fragment contributes to the longer correlation time for proximal domain that has its N terminus intact, while the significant difference of correlation time supports the observation that the two subunits are not restrained and move independently from each other in solution. As expected for independent subunits, the core structure of the two SUMO domains is not affected by conjugation or dimer formation.

### The interaction between SUMO-2 dimers and multiple SIMs

Binding of single SIMs to either SUMO-1 or SUMO-2/3 has been studied in a few cases, such as the SIMs from RanBP2, PIASX, Daxx and MCAF-1 (refs 29, 30, 31, 32, 33, 34). In all of these studies, the SIM peptides consistently bind to a hydrophobic patch between the second β-strand and the following α-helix. The SIM adopts an extended conformation forming the outer most strand, and can be either parallel or antiparallel to the second β-strand.

**Figure 5:**
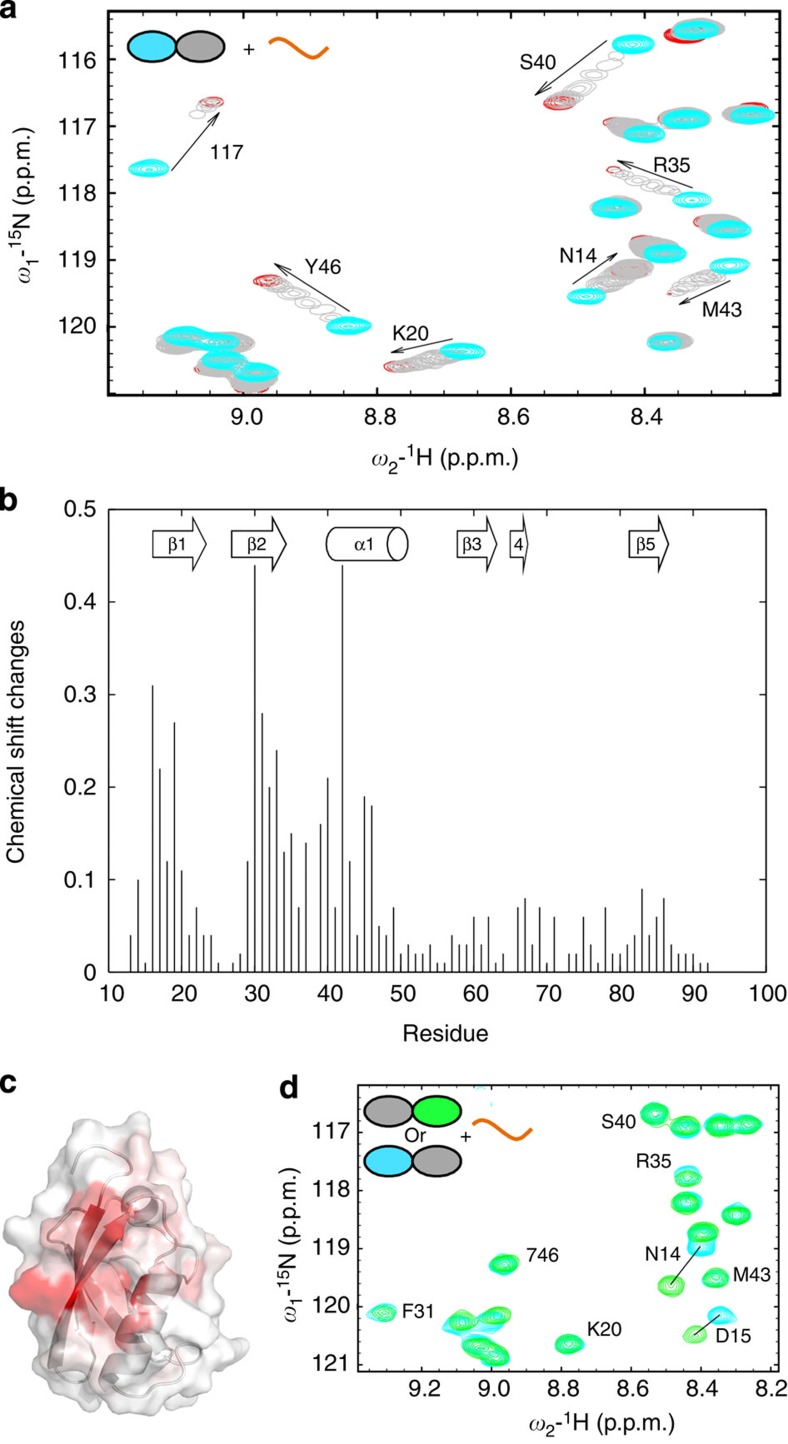
NMR chemical shift mapping of RNF4-SIM2,3 interaction with lys11-linked diSUMO-2. (**a**) ^1^H–^15^N HSQC spectra of SUMO-2 dimer with the distal domain (SUMO-2 ΔN11,cyan) labelled and titrated with increasing molar equivalents of SIM2,3 peptide. Large chemical shift changes in response to increase amount of the SIM2,3 peptide indicate a specific interaction that is fast-intermediate exchange on the NMR timescale. (**b**) Plot of weighted chemical shift perturbations calculated using [(^1^H difference)^2^+((^15^N difference)1/5))^2^]^0.5^ versus residue number for the distal domain; those with changes >0.1 p.p.m. indicate likely proximity to the binding site. (**c**) Map of the significant chemical shift changes (>0.1 p.p.m.) are coloured red on a hybrid cartoon-surface representation of the distal domain structure. This highlights binding of the SIM2,3 peptide to the canonical SIM-binding site between second β2-strand and the α-helix. (**d**) Overlay of ^1^H–^15^N HSQC spectra for distal (cyan) and proximal (green) domains with the SUMO-2 dimer in complex with SIM2,3 shows that very similar chemical shift changes occur for both subunits except for residues close to the isopeptide bond (that is, N14 and D15 shown here). The high degree of similarity between chemical shifts of SIM-binding site residues for both distal and proximal SUMO-2 domains indicate that direction of the SIM2,3 peptide (N- to C terminus) relative to diSUMO-2 chain (distal to proximal) is exchangeable.

Titration of a peptide (SIM2,3) containing SIM2 and SIM3 from RNF4 into SUMO-2 dimers results in significant chemical shifts changes to a number of peaks within the SIM-binding site as suggested by previous studies (Fig. 5a). These residues, namely, N14, H16, I17, N18, L19, K20, V29, Q30, F31, K32, I33, K34, R35, T37, L39, S40, L42, M43, A45 and Y46, as identified from chemical shift changes between the free and bound states (Fig. 5b), are mapped onto the SUMO-2 structure (Fig. 5c). In addition to the second β-strand and the helix, many residues from the first β-strand are also actively involved in the binding. Small chemical shift changes are also observed in many other residues, especially those neighbouring the binding site residues, suggesting subtle structural adjustments induced by SIM binding. Interestingly, titration caused very similar chemical shift changes for both subunits (Fig. 5d), which suggests that the two SIMs bind simultaneously to the SUMO dimer, but exchange SIM-binding partners on a fast timescale, such that each subunit exhibits the same average chemical shift changes in the NMR titration. Furthermore, in the context of tetraSUMO-2 and the full SIM region of RNF4, NMR titrations display the same chemical shift perturbations, confirming that SIM2,3 are the most important binding determinants and conformation exchange still occurs.

### The binding mode of SIM2,3 to diSUMO-2 is bi-directional

**Figure 6:**
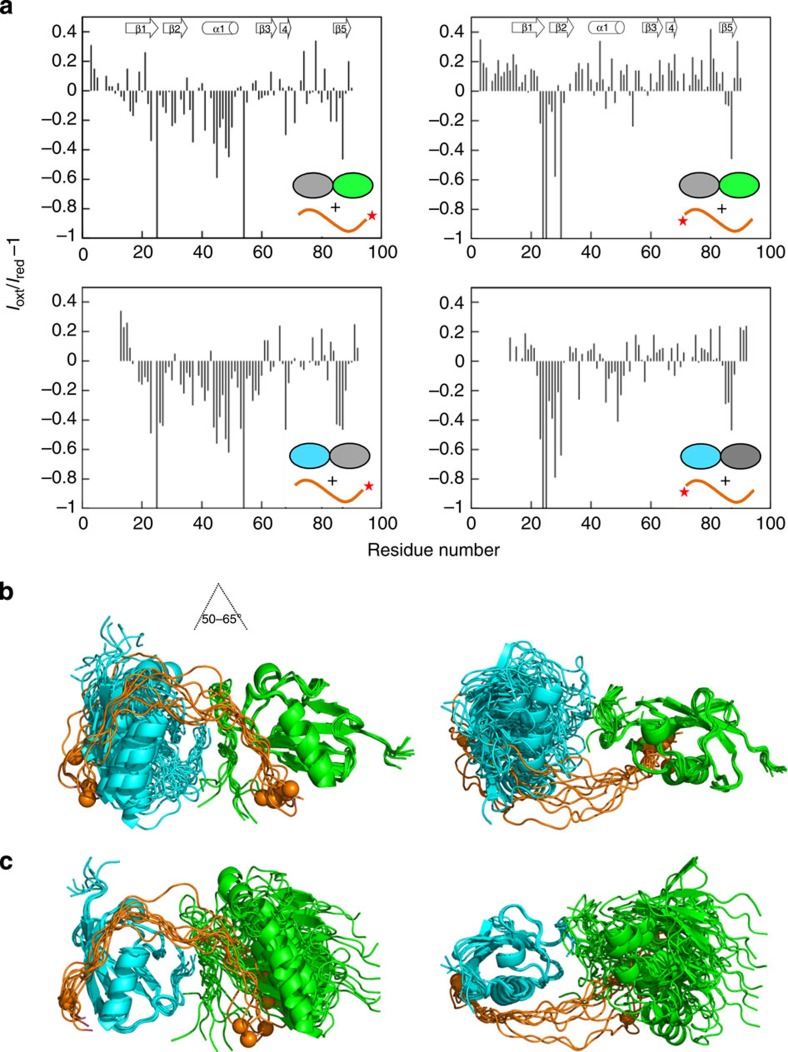
NMR structure of diSUMO-2 in complex with RNF4-SIM2,3. (**a**) Plot of intensity difference between spectra for SUMO-2 dimers in complex with spin-labelled SIM2,3 peptide compared with the spectra in which MTSL is reduced by ascorbic acid. Data for complexes in which the distal subunit SUMO-2 ΔN11 is labelled are presented on the bottom row, whereas those for labelled proximal subunit SUMO-2 ΔGG are shown on the top. Data for complexes with spin-labelled SIM2,3 with MTSL positioned at C terminus are shown on the left and with MTSL at the N terminus on the right. Peak broadening (indicated by an intensity drop) is observed on the canonical SIM-binding site on β2 strand for both domains whether the peptide is N- or C-terminally spin-labelled. This suggests that the binding direction of the SIM2,3 peptide (N- to C terminus) relative to the SUMO chain (distal to proximal) is exchangeable. There are differences within the α-helix whether the C terminus of SIM2,3 is spin-labelled (that is, near SIM3) or at the N terminus (near SIM2). This indicates that the actual binding mode of SIM3 is distinct from SIM2, in which the helix is largely unaffected (right). (**b**) The NMR ensemble of the lowest energy 10 NMR structures for the complex of SUMO-2 dimer with SIM2,3 in which the proximal subunits (SUMO-2 ΔGG) are superposed. (**c**) The NMR ensemble of the lowest energy 10 structures for the complex of SUMO-2 dimer with SIM2,3 in which the distal subunits (SUMO-2 ΔN11) are superposed. The orientations showed on the right represent a 90° rotation from that on the left. The location of the N terminus of the SIM peptide is indicated by a sphere to illustrate its bi-directionality.

To provide further insight into the binding orientations, we introduced the spin label *S*-(2,2,5,5-tetramethyl-2,5-dihydro-1H-pyrrol-3-yl)methyl methanesulfonothioate (MTSL) to either the N- or C terminus of SIM2,3 by mutating a single residue at these positions to cysteine and removing the naturally occurring cysteine within the SIM2,3 linker. The paramagnetic reagent MTSL shortens the transverse relaxation time of nearby nuclei and therefore leads to a loss of peak intensity in the spectrum for distances up to ~20 Å. Titration of either N- or C-terminally spin-labelled SIM2,3 caused peak broadening at specific residues in the SUMO-2 subunits (Fig. 6a). For both domains, a number of broadened residues lie on the β2 strand that interacts directly with the SIM and the β1-β2 loop. In fact, these residues are broadened by the spin-labelled SIM2,3 peptide in all scenarios, that is, for both N- or C-terminally spin-labelled peptides in complexes with SUMO dimers whether the distal or proximal domain was isotope labelled. These data are consistent with a model in which the peptide termini are located at the open end of the SIM-binding sites, and the binding orientation of the peptide is subject to exchange relative to the diSUMO-2 chain direction (that is, proximal to distal). There are, however, marked differences within the α1-helix when comparing data for C-terminally spin-labelled SIM2,3 peptide (that is, near SIM3) with that for the N-terminally labelled one. In the former, a significant number of peaks for residues in α1 are broadened that are unaffected when the label is near SIM2 at the N terminus, indicating that the binding mode of SIM3 is distinct from that observed for SIM2. Spectra recorded after reduction of MTSL by ascorbic acid overlay well onto the spectra with the natural SIM2,3 peptide, indicating that spin labelling does not alter the binding mode.

### The SUMO domains become oriented in the RNF4–SIM complex

Although the chemical shift degeneracy of Lys11-linked SUMO-2 dimers in the complex and lack of NOEs between the two domains point to the absence of a strong inter-domain interaction, we assessed whether the two SUMO domains were restrained by virtue of their interaction with the SIM2,3 peptide. From an analysis of NMR relaxation data for the complex, we derive similar rotational correlation times for the two subunits (9.9±0.2 and 10.2±0.2 ns for the distal SUMO (SUMO-2 ΔN11) and proximal domain (SUMO-2 ΔGG). These are significantly longer than those measured for free Lys11-linked diSUMO-2, which suggests that the two domains bind a single SIM2,3, become ordered and subsequently move as an essentially rigid entity in solution (Supplementary Fig. 5).

To further clarify the relative domain orientation, we measured residual dipolar couplings (RDCs) for the two SUMO domains using Pf1 phage to introduce partial alignment in the solution. RDC measurements revealed alignment tensors for the two domains with similar magnitude, suggesting that they are aligned to similar extent, and likely as a single globular unit. Introduction of these measurements into a structure calculation using CNS supplemented with paramagnetic relaxation enhancements and NOEs did not result in additional violations (Table 1), but defined a partial relative orientation between the two SUMO-2 domains, as shown in Fig. 6b,c. When either the distal or proximal SUMOs are superimposed for the 10 models with lowest energy, the other SUMO-2 subunit is distributed in a much smaller range compared with free diSUMO-2, with only minor deviations in orientation and separation relative to the distal SUMO-2. In the ensemble, the distal SUMO domain is rotated by ~50–65^o^ about an axis perpendicular to the β-sheet of the proximal domain, illustrated on the left orientation shown in Fig. 6b). The relative SUMO orientation observed in our Lys11-linked diSUMO-2/SIM2,3 structure is not seen in any of symmetry neighbours present in crystals of mono SUMO-2 and the linear dimer^38^,^41^. This highlights the challenges in crystallizing SUMO chain complexes with tandem SIMs.

### The SIM2,3 linker contributes to disUMO recognition

**Figure 7:**
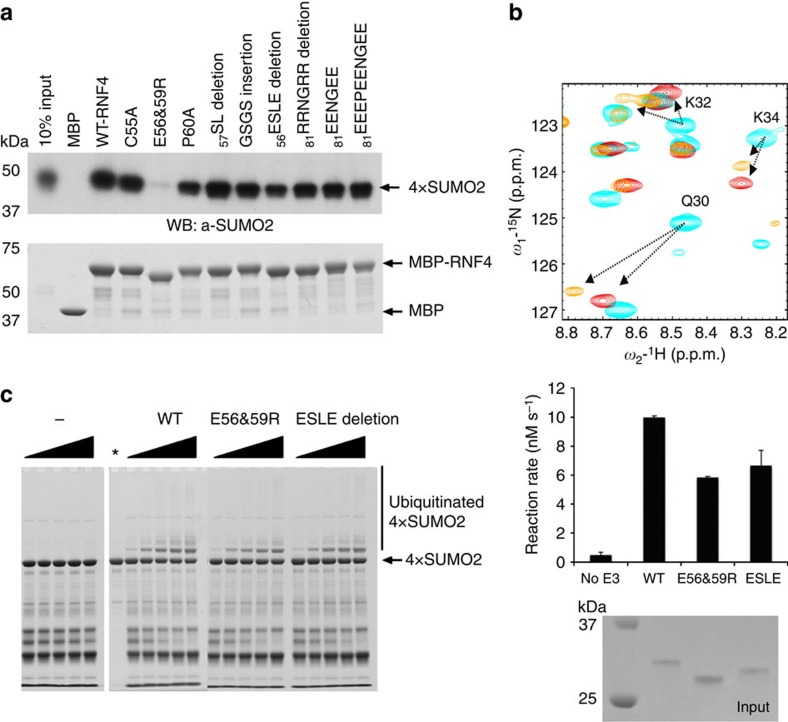
Acidic residues in the SIM2,3 linker contribute to SUMO-2 chain recognition. (**a**) Pull-down experiment with MBP-tagged mutants of RNF4. Bound material was analysed by SDS–PAGE and Coomassie blue staining (lower panel) or by immunostaining with anti-SUMO-2 antibody (upper panel). (**b**) The final point of the ^1^H–^15^N HSQC titration of SUMO-2 dimer with the distal subunit (SUMO-2 ΔN11) labelled with saturating molar equivalents of SIM2,3 peptide and mutant SIM2,3 with E56 and E59 mutated to arginines. The mutant peptide has ~10-fold weaker affinity that is comparable to estimated affinity from mono-SUMO–SIM interactions suggesting that disruption of the linker interactions prevents both SIMs binding the SUMO-2 dimer simultaneously. Differences in the final peak positions indicate the resulting changes in chemical environments of the linker-contacting residues. (**c**) Substrate ubiquitination activity of SIM mutants of RNF4. Coomassie blue-stained SDS–PAGE gel with wild-type (WT) and linker mutant RNF4 proteins (left). Bar chart showing the derived ubiquitination reaction rates for WT and linker mutant RNF4 (right). Reactions were carried out in duplicate and reaction rates are shown as mean with error bars indicating±s.d.

To explore the role of regions outside the SIMs in SUMO interactions, we used the NMR titration experiments to guide our site-directed mutagenesis and tested these mutants for SUMO chain binding and ubiquitination. Pull-down experiments were first carried out using MBP-tagged mutants with tetraSUMO-2 (Fig. 7a). Mutation of the highly charged regions downstream of the SIMs (_81_RRNGRR) showed no appreciable defect in binding polySUMO-2. A measurable reduction in binding was observed for mutations targeting the SIM2,3 linker. Specifically, the P60A mutant and a four residue deletion between SIM2 and SIM3 showed small decreases in bound polySUMO-2, which are consistent with the effects of removing either SIM2 or SIM3 alone (Fig. 2a). In these mutants, the remaining SIMs and the linkages between them enable an appreciable RNF4 interaction to be maintained. An appreciable RNF4-binding defect is only seen when both SIM2 and SIM3 are disrupted, which highlights the importance of their tandem arrangement. Strikingly, a charge reversal of two acidic linker residues (E56R and E59R) almost completely abolished binding (Fig. 7a, lane 4), akin to the defect seen in the double SIM2,3 mutant (Fig. 2a). To investigate this further, a SIM2,3 peptide was synthesized with these charge reversal mutations and the interaction monitored by ^1^H–^15^N HSQC NMR spectra of Lys11-linked diSUMO-2 with an isotope-labelled distal domain (Fig. 7b). The interaction of the mutant peptide is significantly weaker than wild-type with peaks changes showing fast exchange and NMR-estimated dissociation constants for the mutant E56R&E59R–SIM2,3 peptide almost an order of magnitude higher than wild-type peptide and in the same range to those for monomeric SUMO–SIM interactions. These data are consistent with pull-down experiments showing a marked reduction in retained polySUMO (Fig. 7a). We conclude that the charge reversals prevent interactions between the linker and the basic interface between SUMO-2 domains, such that only one SIM can bind efficiently at a time. The charge reversal mutant (E56R&E59R) together with the linker deletion (_56_ESLE) were subsequently tested in single-turnover auto- and substrate- ubiquitination assay. As expected, no defect was observed for autoubiquitination (Supplementary Fig. 6), but the reactions rates for SUMO-targeted ubiquitination by these mutants were significantly reduced (Fig. 7c). Surprisingly, although both mutants remove the acidic nature of the linker and have a detrimental effect on ubiquitination efficiency of SUMO chains, the _56_ESLE deletion shows detectable binding in pull-down assays with tetraSUMO-2. Based on steric considerations of our diSUMO-2/SIM2,3 structure (Fig. 6b,c), it is likely that the short linker of _56_ESLE deletion mutant prevents linear binding of poly SUMO-2, but non-linear arrangements or intermolecular contacts can occur. This observation is consistent with the effect of mutating individual SIMs (Fig. 2). Charge reversal of the SIM2,3 linker (E56R&E59R) introduces significant electrostatic repulsion with the basic interface between SUMO-2 domains while keeping the SIMs properly spaced. This we believe prevents the flanking linker from being accommodated, which has a pronounced effect on the ability of the RNF4–SIMs to interact efficiently with a SUMO-2 chain.

## Discussion

Domain orientation and dynamics has an essential role in the function of multidomain proteins, and the mechanisms of protein–protein recognition for such systems are only recently emerging. For polyubiquitin, the location of the isopeptide linkage determines the overall conformational preferences and flexibility of the chain. Combined NMR-based solution and crystallographic studies have revealed that Lys48-linked ubiquitin chains adopt a compact structure, whereas the conformation of Lys63-linked ubiquitin chains is more dynamic and open^42^,^43^.

In a free Lys11-linked polySUMO-2 chain, solution NMR data revealed a paucity of specific inter-domain interactions; therefore, it is not surprising that no preferred relative orientation of the two SUMO domains is adopted, and these dynamics enable the majority of domain surfaces to be available for interaction. RNF4 recognizes long SUMO-2 chains via an extensive disordered region located at the N terminus of the RING domain, which contains four canonical SIM motifs. In addition to all four SIMs being able to bind SUMO-2 chains, NMR chemical shift analysis suggested that an additional SIM-like sequence (‘SIM5’) and a highly basic region may undergo subtle structural rearrangements upon SUMO-2 binding (Fig. 1a). Despite the prospect for the additional contact points with the polySUMO-2 chain, mutagenesis highlighted the dominant role for the region encompassing the second and the third SIMs (SIM2,3) in binding and the targeted ubiquitination of SUMOylated substrates. In fact, SIM2,3 is sufficient and required for efficient polySUMO-2 recognition and ubiquitination. Furthermore, the central functional role of the tandem SIM2,3 sequence is supported by the ability of RNF4 to ubquitinate SUMO-2 dimers *in vitro*^18^ and more efficiently *in vivo*^38^.

**Figure 8:**
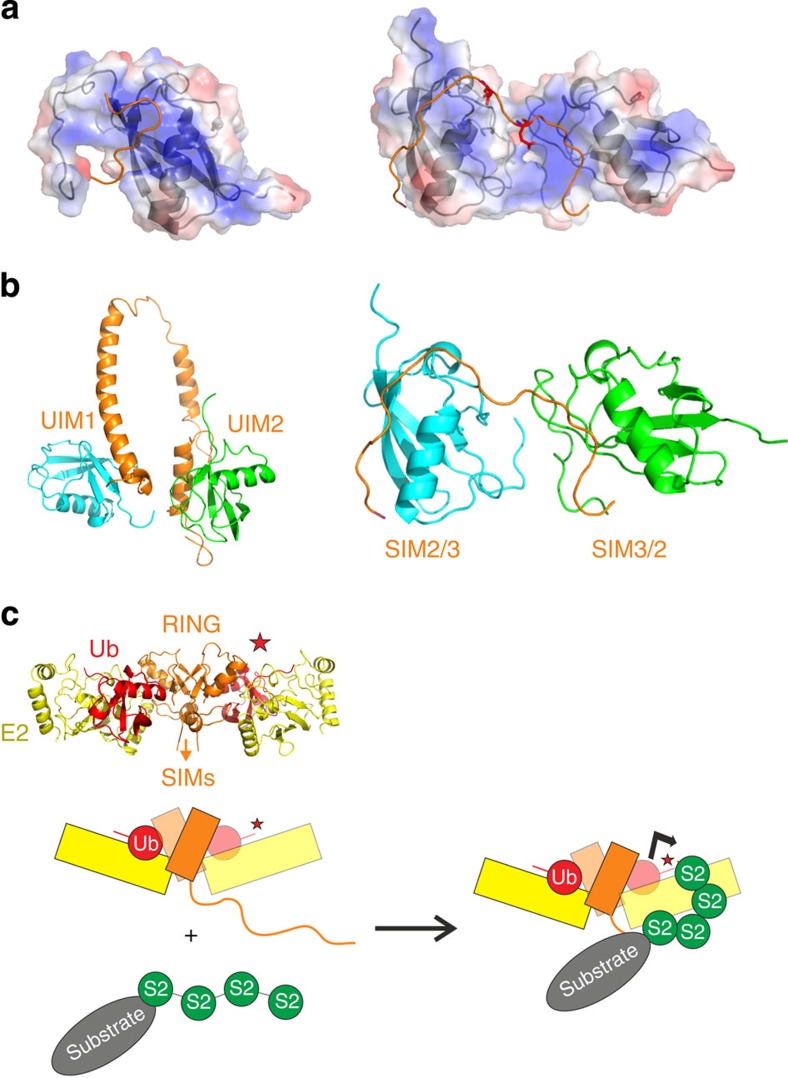
Tandem SIMs manipulate SUMO-2 chain conformation. (**a**) Comparison of the electrostatic surfaces of the solution structure of SUMO-3 in complex with the single SIM_MCAF1_ (2RPQ^31^, left) and our structure of Lys11-linked diSUMO in complex with SIM2,3 from RNF4 (right). SUMO-3 is oriented in the same manner as the distal SUMO-2 domain of the Lys11-linked dimer. The SUMO domain surfaces are shown as partially transparent with positively charge regions coloured blue and negative red, and a grey cartoon representation of the domain structure visible beneath. The path of the SIM regions are shown as an orange ribbon with acidic amino-acid residues outside of the SIMs shown as red sticks. In the monomeric SUMO-3 (left), the acidic sequence C-terminal to the SIM folds back (‘DDEE’ in red), whereas in the SUMO-2 dimer the path of the SIM region continues contiguously to the adjacent SUMO domain. The acidic residues E56 and E59 (red sticks) in the SIM2,3 linker interact within the positively charged cleft (blue surfaces) at the diSUMO-2 interface. (**b**) Cartoon representation for the solution structure of Lys48-linked di-ubiquitin in complex with tandem UIMs of the proteasome adaptor S5a (S5a-UIMs)^45^ (left) alongside a comparable orientation of Lys11 diSUMO with SIM2,3 (right). (**c**) Schematic diagram summarizing how conformational restriction and manipulation of the polySUMO chain by the RNF4 SIM region might promote efficient ubiquitin transfer to polySUMO substrates. As reference, a ribbon representation of X-ray structure of RNF4–RING bound to the ubiquitin-loaded E2 UbcH5A is shown above. Ubiquitin is coloured red and the location of the C-terminal thioester bond indicated by a star for one of the domains. The RNF4 and E2 UbcH5A are coloured orange and yellow, respectively. For clarity, only a single SIM region is shown from one of the RNF4 protomers of the dimer.

Our NMR solution structure of Lys11-linked diSUMO-2 bound to SIM2,3 shows that the two SUMO domains bind simultaneously to these SIMs, which are separated by only eight residues. In comparison with free diSUMO-2, binding of SIM2,3 significantly limits the freedom and severely restricts the relative motion of the SUMO domains. This is consistent with conformation exchange effects seen in the NMR spectra of isopeptide bond resonances, despite their distance from the SIM-binding sites. Crystal structures of monomeric SUMO-1 domains in complex with single SIM regions have been reported previously^28^,^29^,^31^,^34^, and the orientation of the SIMs with respect to β2 edge strand has been observed in either antiparallel (SUMO-1/SIM_RanBP2_) and parallel (SUMO-1/SIM_PIASx_) orientations. Subsequent NMR studies have shown dynamic exchange between parallel and antiparallel orientations for individual SIM sequences binding to SUMO^32^,^44^ and similar exchange is observed in monoSUMO interactions with RNF4–SIMs. Our NMR studies of the diSUMO-2/SIM2,3 complex show that the tandem SIMs can bind to diSUMO-2 chain in either direction (that is, both SIMs can bind to either the distal or proximal SUMO domain). In the context of the bound SUMO-2 domain, SIM2 lies parallel to the second β-strand whether it is interacting with distal or proximal domains, whereas SIM3 is found in the opposite direction (antiparallel to β2). As flanking acidic residues are known to have a role in defining the parallel SIM orientations with monomers^33^,^44^, we postulated that the acidic linker between SIM2,3 together with the additional asymmetry of SIM2 would contribute to this arrangement. A role for electrostatic contributions to SUMO-1 binding is highlighted by the SUMO-1 versus SUMO-2/3 paralogue selectivity upon SIM_DAxx_ phosphorylation^34^. Furthermore, the solution structure of monomeric SUMO-3/SIM_MCAF1_ revealed a flexible ‘DDEE’ region after the SIM sequence that folds back and engages transiently with a positive surface patch on the SUMO monomer (Fig. 8a, left). Although this bears some resemblance to our diSUMO-2/SIM2,3 structure in that acidic residues outside of the SIM sequence contribute, the exact nature of these electrostatic interactions are distinct owing to the juxtaposition of the conjugated SUMO domains. The RNF4–SIM2,3 linker containing two acidic residues, namely E56 and E59, contacts with the SUMO-2 dimer interface and therefore does not fold back but sits within a large, basic groove formed by the arrangement of the two neighbouring SUMO domains (Fig. 8a, right).

The higher affinity of the SIM2 parallel interaction and the reinforcement of this arrangement by the acidic linker explain why SIM2 mutations have the largest effects on RNF4 binding to SUMO-2 chains as well as to ubiquitination. The lysine residues that are ubiquitinated (predominantly K11, but also K32, K41 and K44)^18^ are all located on the same side of the SUMO-2 domain. Owing to the close juxtaposition of the two SUMO-2 domains, these residues on the proximal SUMO-2 are not accessible for conjugation as they are shielded by the distal SUMO-2 domain. Instead, all the lysine residues on the distal SUMO are available for ubiquitination. In addition to the restriction of inter-domain motion in the polySUMO-2 chain, SIM2,3 induces a defined, overall bend in the shape of the complex. This conformational manipulation provides a mechanism to guide the distal SUMO-2 moiety closer to the active thioester intermediate for efficient ubiquitination by RNF4. Furthermore, residual opening–shutting movements of the SUMO-2 dimer would allow some conformational sampling to engage with the RING-activated, ubiquitin-loaded E2. The exchangeable nature of the SIM polypeptide chain direction relative to SUMO-2 domains (that is, distal to proximal or *vice versa*) may explain why ubiquitin transfer by the RNF4 dimer can proceed *in cis* or *in trans*^35^.

An intriguing comparison can be made between our diSUMO-2/SIM2,3 structure and the binding mode observed for Lys48-linked di-ubiquitin in complex with the tandem ubiquitin interaction motifs (UIMs) of the proteasome adaptor S5a (S5a-UIMs)^45^. Although UIMs are short hydrophobic sequences not unlike SIMs, they form a helical interaction with the β-sheet surface of ubiquitin, whereas SIMs adopts an extended conformation and augment the edge of the SUMO β-sheet. A striking analogy is, however, highlighted in di-ubiquitin/S5a-UIMs structure, where the tandem UIMs engage a single di-ubiquitin molecule^45^ (Fig. 8b), but act independently with mono-ubiquitin^46^. The two UIMs bind with different affinity and in a defined overall orientation both with respect to the ubiquitin domain itself and the polyubiquitin chain. Both UIM helices lie parallel to the C-terminal strand of ubiquitin, but UIM2 binds exclusively to the proximal domain, whereas UIM1 is located at the distal end. The S5a-UIMs do not exchange, but can be forced to compete for the distal subunit when the proximal domain is occupied by the adaptor Rpn13. Structural analyses of these two systems point to a common conclusion in that cooperative binding and conformation manipulation of polySUMO/polyubiquitin chain are important for orienting it optimally for processing (Fig. 8c). In the case of RNF4, dimerization is essential for ubiquitin transfer and regulation of thioester intermediate with ubiquitin^35^,^47^. It is therefore conceivable that the conformational manipulation induced by binding the RNF4 SIM region has a key role in drawing longer SUMO-2 chains together (>4 SUMO domains) so that they can engage >1 RNF4 N terminus and stabilize the active RNF4 dimer. Consistent with this hypothesis is recent evidence showing that long SUMO chains induce dimerization and activation of RNF4 (refs 38, 48) that facilitates both substrate ubiquitination and degradation of RNF4 by autoubiquitination. Although crystallographic evidence has illuminated the priming mechanism for ubiquitin transfer^35^,^36^, it has been unable to provide insight into polySUMO-modified substrate delivery to this machinery. Our solution NMR characterization of segmentally labelled polySUMO/RNF4-SIM complexes yields new findings regarding polySUMO chain recognition, dynamics and delivery to the ubiquitin ligation machinery.

## Methods

### Preparation of RNF4 and RNF4 fragments

RNF4 ΔN31 was expressed from pLou3 vector in *Escherichia coli* BL21(DE3) cells and RNF4 32–133 was expressed from pHIS-TEV-30a vector in *E. coli* Rosetta (DE3) cells as described previously^49^,^50^. For NMR, cells were grown in M9 minimal medium at 37 °C supplemented with ^15^NH_4_Cl and ^13^C-glucose (Sigma). Purification by Ni-NTA (Qiagen) chromatography after cleavage with TEV protease was used to remove any uncleaved fusion protein. Proteins were further purified to homogeneity by gel filtration chromatography. SIM peptides characterizing the sequences of the two central SIM motifs from RNF4 were synthesized on an Intavis ResPep SL peptide synthesizer and purified with Liquid Chromatography-Mass Spectrometery (LC-MS) in house. The sequences of SIM2, SIM3 and SIM2,3 are TVGDEIVDLTCES, SLEPVVVDLTHND and TVGDEIVDLTCESLEPVVVDLTHND, respectively. Two peptides with a cysteine residue at the N- and C terminus, respectively, and replacing the internal cysteine with serine, with serine and a peptide with the two glutamates in linker replaced by arginines, were commercially obtained from LifeTein LLC. Site-directed mutagenesis was performed to test the effects of mutations in the SIMs, the SIM2,3 linker and downstream basic region in polySUMO binding and ubiquitination (Supplementary Table 1).

### Preparation of SUMO proteins and segmentally labelled SUMO dimers

SUMO-2 sequences were cloned into pHIS-TEV-30a and transformed into *E. coli* BL21(DE3) cells, which were then grown in LB or M9 media with ^15^NH_4_Cl and ^13^C-glucose as nitrogen and carbon sources for NMR studies.

To overcome the overlap of resonances, only one of the two SUMO-2 domains was labelled with ^13^C and ^15^N, whereas the other was unlabelled. To maximize the yield of SUMO-2 dimer, two SUMO-2 constructs were made, with one lacking the N-terminal 11 residues and the other lacking the C-terminal 2 glycine residues. SUMO-2 proteins were then purified by Ni-NTA chromatography. The conjugation reaction for preparation of SUMO-2 chains contained SUMO-2 (500 μM), GST-tagged SAE1/SAE2 0.26 μM, His6-tagged Ubc9 (16 μM), ATP (3 mM), MgCl_2_ (5 mM), DTT (5 mM) and Tris (pH 7.5, 50 mM). The reaction was incubated at 37 °C for 9–10 h. SAE1/SAE2 and Ubc9 were then removed from the reaction mixture by affinity chromatography. The flow-through was concentrated and loaded on a gel filtration column (HiLoad 16/60 Superdex 75, GE Healthcare) preequilibrated in 20 mM Tris, 150 mM NaCl, 1 mM TCEP, pH 7.5, and polymeric SUMO-2 chains were resolved.

### Interaction analysis using pull-down assays

Analysis of binding between SUMO-2 and various RNF4 constructs was carried out using a pull-down assay. 4 × SUMO-2 (3.7 or 7.4 μM) was mixed with MBP-tagged RNF4 (1.2 μM) and 10 μl of amylose beads in 50 mM Tris, 150 mM NaCl, 0.5 mM TCEP, 5% (v/v) glycerol, 0.1% NP40, pH 7.5, in total volume of 60 μl. Bound proteins were eluted with SDS–PAGE loading buffer and analysed by western blotting with 1:1,000 anti-SUMO-2 antibody, which was prepared in house as described previously^18^

### Single-turnover ubiquitination assay

In single-turnover ubiquitination assays, E2 (UbcH5a) was first charged with ubiquitin in the absence of E3 and substrate. To prepare UbcH5a~Ub thioester, UbcH5a and ubiquitin (both 100 μM) were incubated with 0.2 μM Uba1 in 50 mM Tris, 150 mM NaCl, 3 mM ATP, 5 mM MgCl_2_, 0.5 mM TCEP, 0.1% NP40, pH 7.5, at 37 °C for 12 min. To stop E1-mediated loading of E2 with ubiquitin, ATP was depleted by addition of apyrase (4.5 U ml^−1^; New England BioLabs) and the reaction was incubated at room temperature for 10 min. In an autoubiquitination assay, UbcH5a was charged with ^125^I-labelled ubiquitin (~320 Ci mol^−1^). UbcH5a~^125^I-Ub (~20 μM) was then mixed with RNF4 (0.275 μM) and incubated at room temperature. In a substrate ubiquitination assay, ^125^I-labelled 4 × SUMO-2 (~750 Ci mol^−1^) was used as a substrate for RNF4-mediated ubiquitination. UbcH5a~Ub thioester (~20 μM) was incubated with RNF4 (0.275 μM) and 5.5 μM ^125^I-4 × SUMO-2 at room temperature. Reactions were stopped by analysis using SDS–PAGE buffer, and analysed by phosphorimaging (FujiFilm FLA-5100). Reaction rates were determined using at least three time points within the linear range of the reaction. Reactions were carried out in duplicate, and reaction rates are shown as mean±s.d.

### NMR resonance assignment and structure calculation

SUMO dimers were dissolved in 300 μl NMR buffer containing 20 mM Tris–HCl, pH 7.0, 100 mM NaCl, 2 mM TCEP and 10% D_2_O to a final concentration of ~300 μM. A full set of triple resonance experiments including HNCACB, CBCACONH, HNCO and HN(CA)CO were recorded for backbone assignments, whereas HBHACONH, HCC(CO)NH-TOCSY, HC(C)H-TOCSY and (H)CCH-TOCSY were recorded for side-chain assignments. Peaks picked from 3D ^15^N/^13^C-edited NOESY spectra together with dihedral angle restraints derived from TALOS+ were used in ARIA to calculate the SUMO structures in the dimers^39^,^40^,^51^. Similar procedures were followed after formation of complexes between SUMO dimers and the SIM peptides, to assign chemical shifts and calculate the SUMO structures in the complex environment. Combined chemical shift perturbations were calculated using [(^1^H difference)^2^+((^15^N difference)1/5))^2^]^0.5^.

### Measurement of NMR relaxation times, paramagnetic relaxation enhancements and RDCs

^15^N T_1_, T_2_ parameters were measured for both SUMO-2 domains in free and complex states^52^. The model-free approach was used to derive rotational correlation times for each data set^53^. For spin labelling, the free cysteine of the SIM peptide was covalently bonded to MTSL^50^,^54^. An amount of 1.7 mg of peptide was dissolved in 450 μl buffer and 5 mg of spin label was subsequently added in 50 μl DMSO solution. The mixture was incubated overnight at 277 K and washed using a concentrator to remove unreacted reagents. ^1^H–^15^N HSQC spectra of segmentally labelled SUMO-2 dimer were recorded in the presence of saturating amounts of spin-labelled SIM peptide. The paramagnetic effect was removed by reduction with ascorbic acid, and a final HSQC recorded. RDCs^54^,^55^ were measured using In-phase Anti-phase (IPAP) experiments^56^ with samples containing 14 mg ml^−1^ Pf1 phage. The alignment tensor was obtained using PALES^57^ and then used in CNS^58^ for modelling of the complex structure.

## Author contributions

R.T.H., S.J.M., A.P. and P.S. conceived and designed the study; NMR experiments were designed by S.J.M., P.S. J.M. and Y.X.; P.S., S.K., Y.X. and A.P. performed the NMR experiments and data analysis; and A.P. and O.L. carried out cloning, protein purification, *in vitro* binding assays, ubiquitination assays and isothermal calorimetry. All authors contributed the manuscript preparation.

## Additional information

**Accession codes**: The coordinates for the 10 lowest energy structures of the complex between lys11 linker SUMO-2 dimer and RNF4-SIM2,3 have been deposited in the Protein Data Bank with PDB ID code 2mp2 with associated NMR assignments under the BRMB ID 19961.

**How to cite this article:** Xu, Y. *et al*. Structural insight into SUMO chain recognition and manipulation by the ubiquitin ligase RNF4. *Nat. Commun.* 5:4217 doi: 10.1038/ncomms5217 (2014).

## Supplementary Material

Supplementary Information Supplementary Figures 1-6 and Supplementary Table 1

## Figures and Tables

**Table 1:** NMR structure calculation statistics.

	Subunit 1	Subunit 2	complex
*NMR constraints*			
Unambiguous NOE	1,002	1,153	2,155
Intra-residue	449	427	876
Sequential (*i*−*j*=1)	163	204	367
Medium-range (*i*−*j* <5)	108	146	254
Long range	282	376	658
Ambiguous NOE	559	621	1,180
Ambiguous intermolecular restraints			54
Dihedral angle restraints	78	76	154
Residual dipolar coupling restraints			141
			
*Structure statistics*			
* *Violations			
Number of dihedral angle violation>5°	0	0	3.2±0.7
Number of distance constraint violation >0.5 Å	0	0	2.6±0.9
Number of RDC violation >1 Hz			46±4
Deviation from idealized geometry			
Bond lengths (Å)	0.0047	0.0046	0.0065
Bond angles (°)	0.5747	0.5916	0.798
Average pairwise r.m.s.d for heavy atoms within secondary structures (Å)	0.50±0.07	0.44±0.05	

RDC, residual dipolar coupling; r.m.s.d., root mean squared deviation.

## References

[b1] Weissman A. M. Themes and variations on ubiquitylation. Nat. Rev. Mol. Cell. Biol. 2, 169–178 (2001). 11265246 10.1038/35056563

[b2] Hershko A. & Ciechanover A. The ubiquitin system. Annu. Rev. Biochem. 67, 425–479 (1998). 9759494 10.1146/annurev.biochem.67.1.425

[b3] Schnell J. D. & Hicke L. Non-traditional functions of ubiquitin and ubiquitin-binding proteins. J. Biol. Chem. 278, 35857–35860 (2003). 12860974 10.1074/jbc.R300018200

[b4] Kerscher O., Felberbaum R. & Hochstrasser M. inAnnual Review of Cell and Developmental Biology Vol. 22, 159–180Annual Reviews (2006). 10.1146/annurev.cellbio.22.010605.093503 16753028

[b5] Mahajan R., Delphin C., Guan T. L., Gerace L. & Melchior F. A small ubiquitin-related polypeptide involved in targeting RanGAP1 to nuclear pore complex protein RanBP2. Cell 88, 97–107 (1997). 9019411 10.1016/s0092-8674(00)81862-0

[b6] Hay R. T. SUMO: a history of modification. Mol. Cell 18, 1–12 (2005). 15808504 10.1016/j.molcel.2005.03.012

[b7] Johnson E. S. Protein modification by SUMO. Annu. Rev. Biochem. 73, 355–382 (2004). 15189146 10.1146/annurev.biochem.73.011303.074118

[b8] Rodriguez M. S., Dargemont C. & Hay R. T. SUMO-1 conjugation *in vivo* requires both a consensus modification motif and nuclear targeting. J. Biol. Chem. 276, 12654–12659 (2001). 11124955 10.1074/jbc.M009476200

[b9] Ikeda F. & Dikic I. Atypical ubiquitin chains: new molecular signals - 'Protein modifications: Beyond the Usual Suspects' review series. EMBO Rep. 9, 536–542 (2008). 18516089 10.1038/embor.2008.93 PMC2427391

[b10] Matic I. . *In vivo* identification of human small ubiquitin-like modifier polymerization sites by high accuracy mass spectrometry and an *in vitro* to *in vivo* strategy. Mol. Cell Proteomics 7, 132–144 (2008). 17938407 10.1074/mcp.M700173-MCP200 PMC3840926

[b11] Tatham M. H. . Polymeric chains of SUMO-2 and SUMO-3 are conjugated to protein substrates by SAE1/SAE2 and Ubc9. J. Biol. Chem. 276, 35368–35374 (2001). 11451954 10.1074/jbc.M104214200

[b12] Pickart C. M. & Fushman D. Polyubiquitin chains: polymeric protein signals. Curr. Opin. Chem. Biol. 8, 610–616 (2004). 15556404 10.1016/j.cbpa.2004.09.009

[b13] Eddins M. J., Varadan R., Flushman D., Pickart C. M. & Wolberger C. Crystal structure and solution NMR studies of Lys48-linked tetraubiquitin at neutral pH. J. Mol. Biol. 367, 204–211 (2007). 17240395 10.1016/j.jmb.2006.12.065

[b14] Hurley J. H., Lee S. & Prag G. Ubiquitin-binding domains. Biochem. J. 399, 361–372 (2006). 17034365 10.1042/BJ20061138 PMC1615911

[b15] Deng L. . Activation of the I kappa B kinase complex by TRAF6 requires a dimeric ubiquitin-conjugating enzyme complex and a unique polyubiquitin chain. Cell 103, 351–361 (2000). 11057907 10.1016/s0092-8674(00)00126-4

[b16] Thrower J. S., Hoffman L., Rechsteiner M. & Pickart C. M. Recognition of the polyubiquitin proteolytic signal. EMBO J. 19, 94–102 (2000). 10619848 10.1093/emboj/19.1.94 PMC1171781

[b17] Ulrich H. D. The fast-growing business of SUMO chains. Mol. Cell 32, 301–305 (2008). 18995828 10.1016/j.molcel.2008.10.010

[b18] Tatham M. H. . RNF4 is a poly-SUMO-specific E3 ubiquitin ligase required for arsenic-induced PML degradation. Nat. Cell. Biol. 10, 538–546 (2008). 18408734 10.1038/ncb1716

[b19] Xie Y. . The yeast Hex3.Slx8 heterodimer is a ubiquitin ligase stimulated by substrate sumoylation. J. Biol. Chem. 282, 34176–34184 (2007). 17848550 10.1074/jbc.M706025200

[b20] Sun H., Leverson J. D. & Hunter T. Conserved function of RNF4 family proteins in eukaryotes: targeting a ubiquitin ligase to SUMOylated proteins. EMBO J. 26, 4102–4112 (2007). 17762864 10.1038/sj.emboj.7601839 PMC2230674

[b21] Prudden J. . SUMO-targeted ubiquitin ligases in genome stability. EMBO J. 26, 4089–4101 (2007). 17762865 10.1038/sj.emboj.7601838 PMC2230673

[b22] Cheng C. H. . SUMO modifications control assembly of synaptonemal complex and polycomplex in meiosis of Saccharomyces cerevisiae. Genes Dev. 20, 2067–2081 (2006). 16847351 10.1101/gad.1430406 PMC1536058

[b23] Lin F. M., Lai Y. J., Shen H. J., Cheng Y. H. & Wang T. F. Yeast axial-element protein, Red1, binds SUMO chains to promote meiotic interhomologue recombination and chromosome synapsis. EMBO J. 29, 586–596 (2010). 19959993 10.1038/emboj.2009.362 PMC2789940

[b24] Zhang X. D. . SUMO-2/3 modification and binding regulate the association of CENP-E with kinetochores and progression through mitosis. Mol. Cell 29, 729–741 (2008). 18374647 10.1016/j.molcel.2008.01.013 PMC2366111

[b25] Lescasse R., Pobiega S., Callebaut I. & Marcand S. End-joining inhibition at telomeres requires the translocase and polySUMO-dependent ubiquitin ligase Uls1. EMBO J. 32, 805–815 (2013). 23417015 10.1038/emboj.2013.24 PMC3604719

[b26] Sun H. Y. & Hunter T. Poly-small ubiquitin-like modifier (PolySUMO)-binding Proteins Identified through a string search. J. Biol. Chem. 287, 42071–42083 (2012). 23086935 10.1074/jbc.M112.410985 PMC3516753

[b27] Kerscher O. SUMO junction-what's your function? New insights through SUMO-interacting motifs. EMBO Rep. 8, 550–555 (2007). 17545995 10.1038/sj.embor.7400980 PMC2002525

[b28] Reverter D. & Lima C. D. Insights into E3 ligase activity revealed by a SUMO-RanGAP1-Ubc9-Nup358 complex. Nature 435, 687–692 (2005). 15931224 10.1038/nature03588 PMC1416492

[b29] Song J., Zhang Z. M., Hu W. D. & Chen Y. Small ubiquitin-like modifier (SUMO) recognition of a SUMO binding motif - A reversal of the bound orientation. J. Biol. Chem. 280, 40122–40129 (2005). 16204249 10.1074/jbc.M507059200

[b30] Song J., Durrin L. K., Wilkinson T. A., Krontiris T. G. & Chen Y. A. Identification of a SUMO-binding motif that recognizes SUMO-modified proteins. Proc. Natl Acad. Sci. USA 101, 14373–14378 (2004). 15388847 10.1073/pnas.0403498101 PMC521952

[b31] Sekiyama N. . Structure of the small ubiquitin-like modifier (SUMO)-interacting motif of MBD1-containing chromatin-associated factor 1 bound to SUMO-3. J. Biol. Chem. 283, 35966–35975 (2008). 18842587 10.1074/jbc.M802528200

[b32] Namanja A. T. . Insights into high affinity small ubiquitin-like modifier (SUMO) recognition by SUMO-interacting motifs (SIMs) revealed by a combination of NMR and peptide array analysis. J. Biol. Chem. 287, 3231–3240 (2012). 22147707 10.1074/jbc.M111.293118 PMC3270977

[b33] Hecker C. M., Rabiller M., Haglund K., Bayer P. & Dikic I. Specification of SUMO1- and SUMO2-interacting motifs. J. Biol. Chem. 281, 16117–16127 (2006). 16524884 10.1074/jbc.M512757200

[b34] Chang C. C. . Structural and functional roles of Daxx SIM phosphorylation in SUMO Para log-selective binding and apoptosis modulation. Mol. Cell. 42, 62–74 (2011). 21474068 10.1016/j.molcel.2011.02.022

[b35] Plechanovova A. . Mechanism of ubiquitylation by dimeric RING ligase RNF4. Nat. Struct. Mol. Biol. 18, 1052–1059 (2011). 21857666 10.1038/nsmb.2108 PMC3326525

[b36] Plechanovova A., Jaffray E. G., Tatham M. H., Naismith J. H. & Hay R. T. Structure of a RING E3 ligase and ubiquitin-loaded E2 primed for catalysis. Nature 489, 115–120 (2012). 22842904 10.1038/nature11376 PMC3442243

[b37] Panchal S. C., Bhavesh N. S. & Hosur R. V. Improved 3D triple resonance experiments, HNN and HN(C)N, for H-N and N-15 sequential correlations in (C-13, N-15) labeled proteins: application to unfolded proteins. J. Biomol. NMR 20, 135–147 (2001). 11495245 10.1023/a:1011239023422

[b38] Keusekotten K. . Multivalent interactions of the SUMO-interaction motifs in RING finger protein 4 determine the specificity for chains of the SUMO. Biochem. J. 457, 207–214 (2014). 24151981 10.1042/BJ20130753 PMC3901395

[b39] Rieping W. . ARIA2: automated NOE assignment and data integration in NMR structure calculation. Bioinformatics 23, 381–382 (2007). 17121777 10.1093/bioinformatics/btl589

[b40] Shen Y., Delaglio F., Cornilescu G. & Bax A. TALOS plus: a hybrid method for predicting protein backbone torsion angles from NMR chemical shifts. J. Biomol. NMR 44, 213–223 (2009). 19548092 10.1007/s10858-009-9333-z PMC2726990

[b41] Huang W.-C., Ko T.-P., Li S. S. L. & Wang A. H. J. Crystal structures of the human SUMO-2 protein at 1.6 Å and 1.2 Å resolution. Eur. J. Biochem. 271, 4114–4122 (2004). 15479240 10.1111/j.1432-1033.2004.04349.x

[b42] Varadan R. . Solution conformation of Lys63-linked di-ubiquitin chain provides clues to functional diversity of polyubiquitin signaling. J. Biol. Chem. 279, 7055–7063 (2004). 14645257 10.1074/jbc.M309184200

[b43] Eddins M. J., Varadan R., Fushman D., Pickart C. M. & Wolberger C. Crystal structure and solution NMR studies of Lys48-linked tetraubiquitin at neutral pH. J. Mol. Biol. 367, 204–211 (2007). 17240395 10.1016/j.jmb.2006.12.065

[b44] Escobar-Cabrera E. . Characterizing the N- and C-terminal small ubiquitin-like modifier (SUMO)-interacting motifs of the scaffold protein DAXX. J. Biol. Chem. 286, 19816–19829 (2011). 21383010 10.1074/jbc.M111.231647 PMC3103359

[b45] Zhang N. . Structure of the s5a:k48-linked diubiquitin complex and its interactions with rpn13. Mol. Cell. 35, 280–290 (2009). 19683493 10.1016/j.molcel.2009.06.010 PMC2748877

[b46] Wang Q., Young P. & Walters K. J. Structure of S5a bound to monoubiquitin provides a model for polyubiquitin recognition. J. Mol. Biol. 348, 727–739 (2005). 15826667 10.1016/j.jmb.2005.03.007

[b47] Liew C. W., Sun H., Hunter T. & Day C. L. RING domain dimerization is essential for RNF4 function. Biochem. J. 431, 23–29 (2010). 20681948 10.1042/BJ20100957 PMC3104014

[b48] Rojas-Fernandez A. . SUMO chain-induced dimerization activates RNF4. Mol. Cell. 53, 880–892 (2014). 24656128 10.1016/j.molcel.2014.02.031 PMC3991395

[b49] Bruderer R. . Purification and identification of endogenous polySUMO conjugates. EMBO Rep. 12, 142–148 (2011). 21252943 10.1038/embor.2010.206 PMC3049431

[b50] Battiste J. L. & Wagner G. Utilization of site-directed spin labeling and high-resolution heteronuclear nuclear magnetic resonance for global fold determination of large proteins with limited nuclear overhauser effect data. Biochemistry 39, 5355–5365 (2000). 10820006 10.1021/bi000060h

[b51] Marchant J. . Galactose recognition by the apicomplexan parasite Toxoplasma gondii. J. Biol. Chem. 287, 16720–16733 (2012). 22399295 10.1074/jbc.M111.325928 PMC3351351

[b52] Yip G. N. B. & Zuiderweg E. R. P. Improvement of duty-cycle heating compensation in NMR spin relaxation experiments. J. Magn. Reson. 176, 171–178 (2005). 16009587 10.1016/j.jmr.2005.06.003

[b53] Xia J., Deng N.-j. & Levy R. M. NMR relaxation in proteins with fast internal motions and slow conformational exchange: model-free framework and Markov state simulations. J. Phys. Chem. B 117, 6625–6634 (2013). 23638941 10.1021/jp400797y PMC3727231

[b54] Tzeng S.-R., Pai M.-T. & Kalodimos C. inProtein NMR Techniques Vol. 831, eds Shekhtman A., Burz D. S. 133–140Humana Press (2012).

[b55] Hansen M. R., Mueller L. & Pardi A. Tunable alignment of macromolecules by filamentous phage yields dipolar coupling interactions. Nat. Struct. Biol. 5, 1065–1074 (1998). 9846877 10.1038/4176

[b56] Ottiger M., Delaglio F. & Bax A. Measurement of J and dipolar couplings from simplified two-dimensional NMR spectra. J. Magn. Reson. 131, 373–378 (1998). 9571116 10.1006/jmre.1998.1361

[b57] Zweckstetter M. & Bax A. Prediction of sterically induced alignment in a dilute liquid crystalline phase: aid to protein structure determination by NMR. J. Am. Chem. Soc. 122, 3791–3792 (2000).

[b58] Brunger A. T. Version 1.2 of the crystallography and NMR system. Nat. Protoc. 2, 2728–2733 (2007). 18007608 10.1038/nprot.2007.406

