# Supplementary material for: Structural insight into SUMO chain recognition and manipulation by the ubiquitin ligase RNF4
Source: Nat Commun. 2014 Jun 27;5:4217. doi: 10.1038/ncomms5217 (PMC4083429; doi:10.1038/ncomms5217)
Supplement: Supplementary Information — Supplementary Figures 1-6 and Supplementary Table 1 [file ncomms5217-s1.pdf]

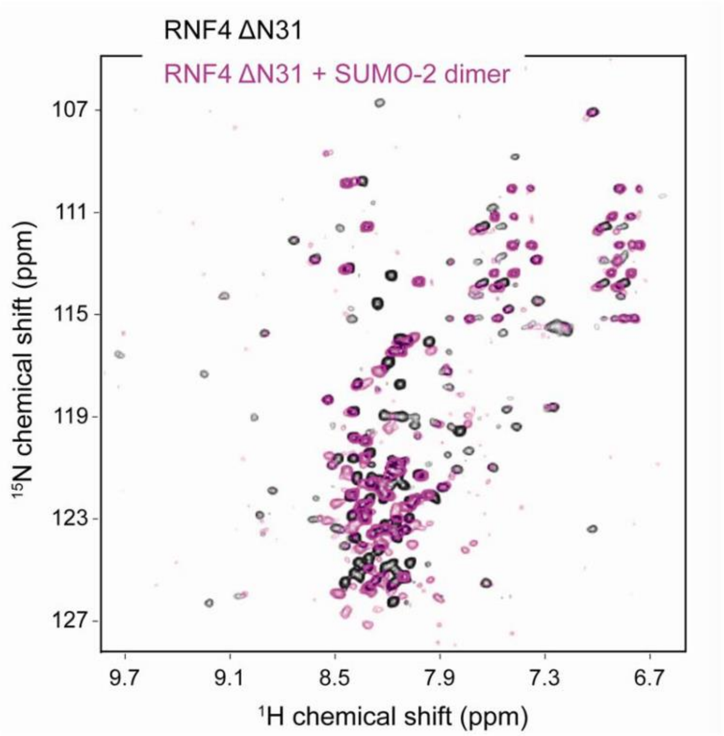

Supplementary Figure 1: Interaction of the SIM region of RNF4 with Lys-linked SUMO-2 dimer. The ^{1}H-^{15}N HSQC NMR spectrum for ^{15}N-^{13}C-labelled RNF4 ΔN31 in the absence (black) and presence of 3.5 molar equivalents of SUMO-2 dimer (magenta). Addition of SUMO-2 dimer results in significant changes in the spectrum, with many resonances shifting or disappearing.

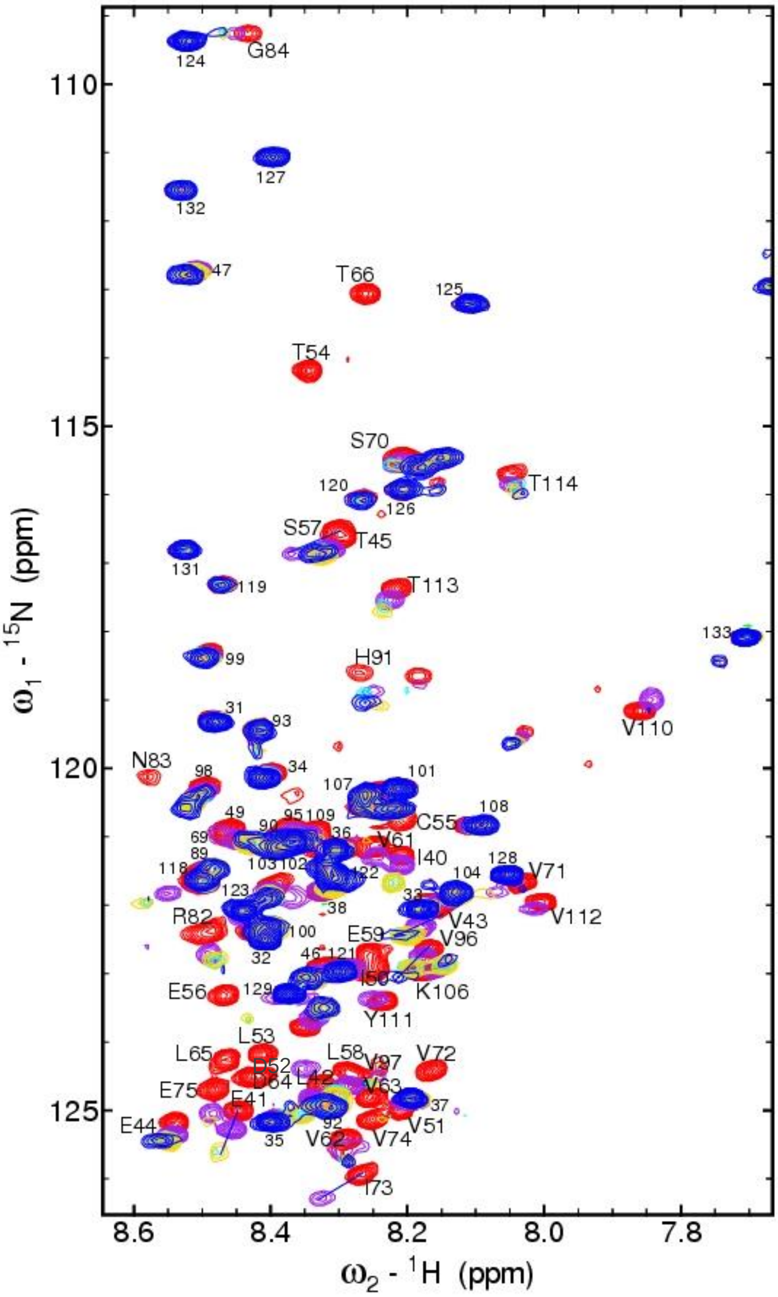

Supplementary Figure 2: NMR resonance assignment of RNF4 32-133. The ^{1}H-^{15}N HSQC spectrum for ^{15}N-^{13}C-labelled RNF4 32-133 (red) overlaid with SUMO titrations (magenta for monomer, cyan for full-length dimer, yellow for truncated dimer, blue for polySUMO chains).

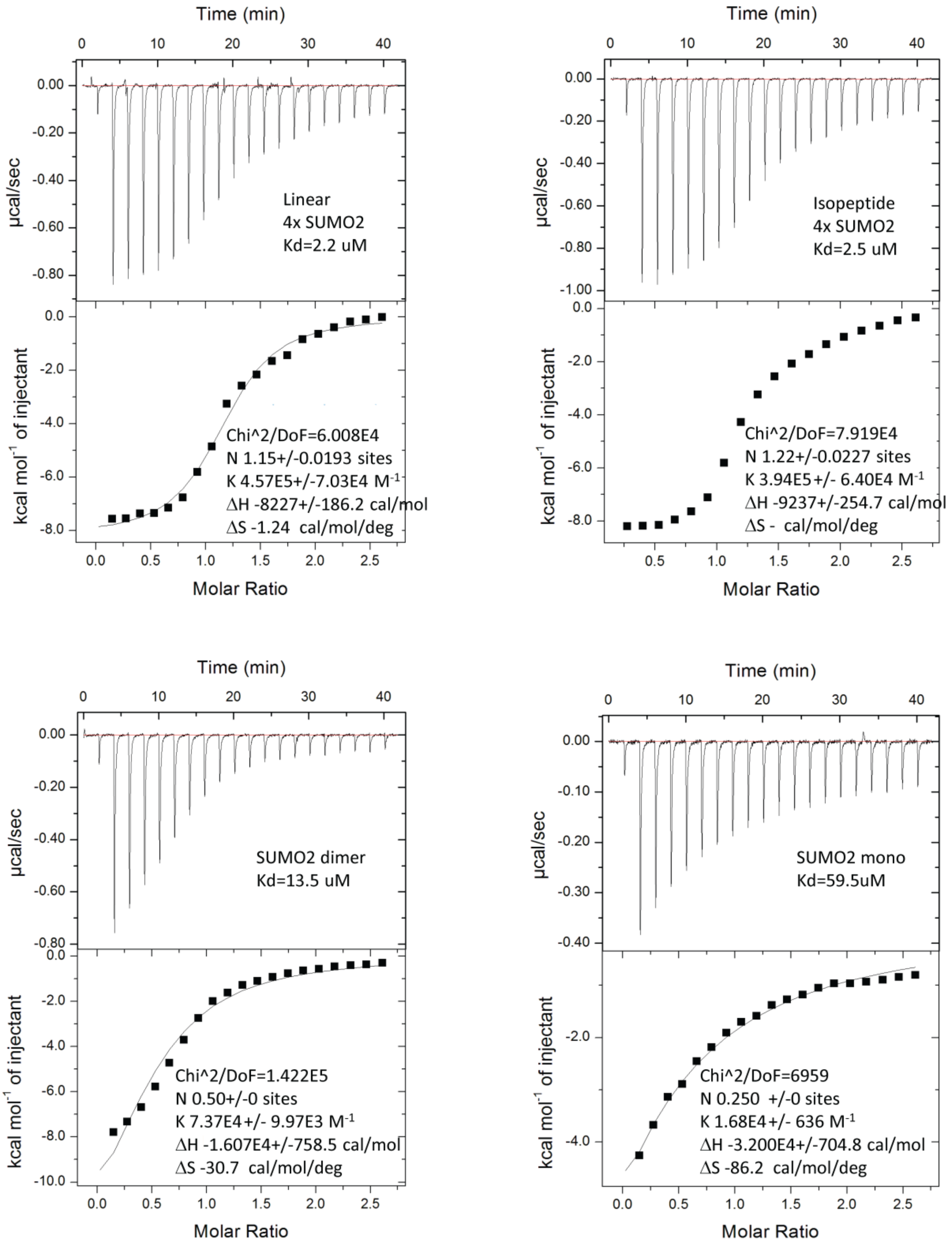

Supplementary Figure 3: Isothermal Calorimetry measurements for the interaction of RNF4 with SUMO chains. Isothermal calorimetric titration of linear tetra-SUMO-2 (top left), isopeptide linked tetra-SUMO-2 (top right), isopeptide linked di-SUMO2 and mono-SUMO-2 into MBP-RNF4 and derived fits.

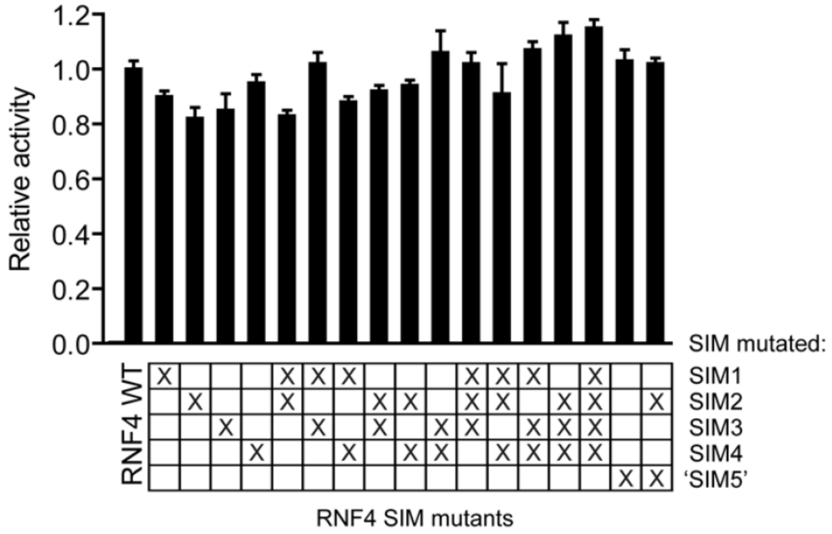

Supplementary Figure 4: Autoubiquitylation activity of SIM mutants of RNF4. Reaction components were resolved by SDS-PAGE and visualized by phosphorimaging. For quantification of autoubiquitylation activity of RNF4, the percentage of conjugated 125I-ubiquitin was determined. Data represent the mean of duplicate reactions with errors displayed as ± standard deviation.

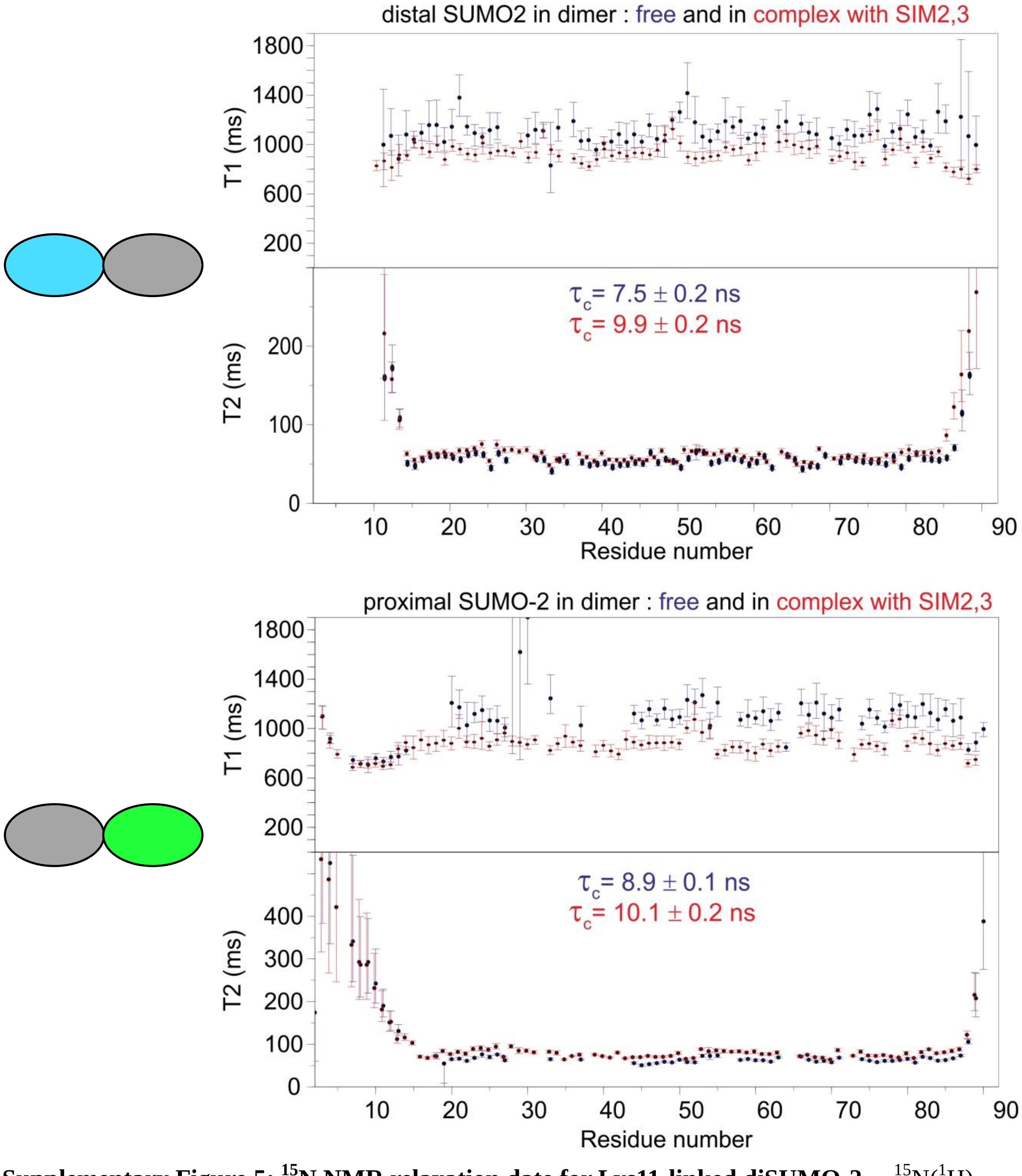

Supplementary Figure 5: ^{15}N NMR relaxation date for Lys11-linked diSUMO-2. ^{15}N(^{1}H) NMR relaxation time as a function of residue number for each SUMO-2 domain of the di-SUMO-2 is its free state (blue) and bound to SIM2,3 (red). Peak heights were fitted to a single exponential decay function and errors derived are standard deviations derived from fitting the data. Calculated rotational correlation times are also shown. The two SUMO-2 domains tumble independently in free isopeptide-linked SUMO dimer, but are not only higher but become near equal in the complex, indicate that they move as a larger, single species.

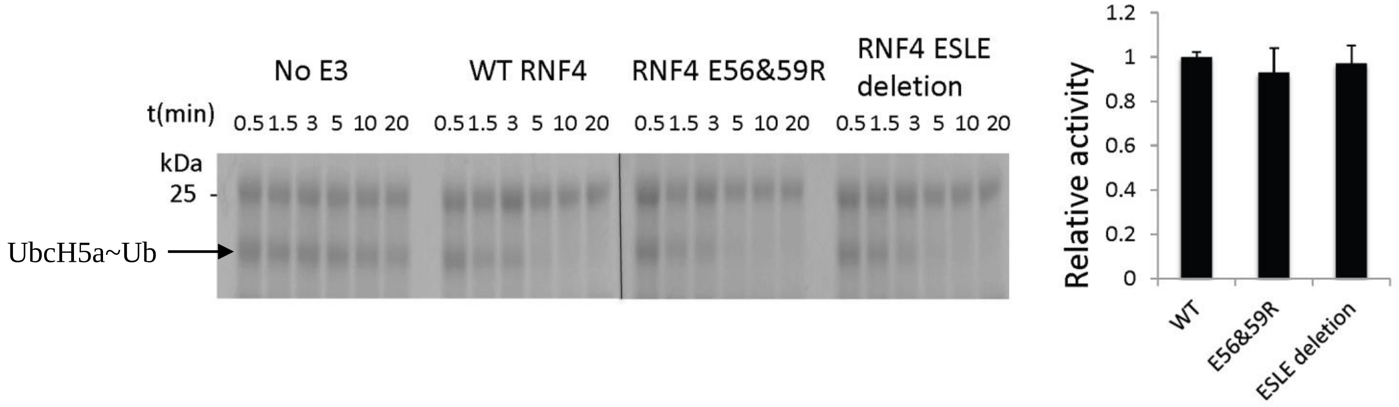

Supplementary Figure 6: Autoubiquitylation activity of SIM2,3 linker mutants of RNF4. RNF4 (0.275 μM) was incubated with ~20 μM UbcH5a~Ub thioester at room temperature for the indicated time period, followed by addition of SDS-PAGE loading buffer. Reaction components were resolved by SDS-PAGE and visualized by staining with Coomassie blue. For quantification of autoubiquitylation activity of RNF4, the percentage of conjugated ubiquitin (as determined from the UbcH5a~Ub levels – lower band) was determined. Data represent mean ± standard deviation of duplicate reactions.

Supplementary Table 1

List of primers for mutagenesis of RNF4-SIM region	
I36A, L38A, V39A forward	GATCTCCTTGGAAGCAGAACCCGCAGAAGCCGCGGAAACTGCTGGAGATG
I36A, L38A, V39A reverse	CATCTCCAGCAGTTTCCGCGGCTTCTGCGGGTTCTGCTTCCAAGGAGATC
I46A, V47A, L49A forward	GAAACTGCTGGAGATGAAGCTGCGGACGCCACTTGTGAATCTTTAGAG
I46A, V47A, L49A reverse	CTCTAAAGATTCACAAGTGGCGTCCGCAGCTTCATCTCCAGCAGTTTC
V58A, V59A, L61A forward	CTTGTGAAT CTTTAGAGCCTGTGGCGGCTGATGCGACTCACAATGACTC
V58A, V59A, L61A reverse	GAGTCATTGTGAGTCGCATCAGCCGCCACAGGCTCTAAAGATTCACAAG
V67A, V68A, I69A, V70A forward	GCGACTCACAATGACTCTGCTGCGGCTGCTGACGAAAGAAGAAGACCAAGG
V67A, V68A, I69A, V70A reverse	CCTTGGTCTTCTTCTTTCGTCAGCAGCCGCAGCAGAGTCATTGTGAGTCGC
V110A, Y111A, V112A forward	GAACTGTCTAAGGACAAAGATGCAGCCGCGACTACCCATACTCCTCG
V110A, Y111A, V112A reverse	CGAGGAGTATGGGTAGTCGCGGCTGCATCTTTGTCCTTAGACAGTTC
C55A forward	GAAATCGTGGACCTCACCGCGGAATCTTTAGAGCCTGTG
C55A reverse	CACAGGCTCTAAAGATTCCGCGGTGAGGTCCACGATTTC
E56R&E59R forward	GACCTCACCTGTCGTTCTTTACGTCCTGTGGTTGTGGAC.
E56R&E59R reverse	GTCCACAACCACAGGACGTAAAGAACGACAGGTGAGGTC
P60A forward	CTGTGAATCTTTAGAGGCGGTGGTTGTGGACCTGAC
P60A reverse	GTCAGGTCCACAACCACGTCAGGTCCACAACCACG
RNF4 SL del forward	CTCACCTGTGAAGAGCCTGTGGTTG
RNF4 SL del reverse	CAACCACAGGCTCTTCACAGGTGAG
GSGS ins forward	CTCACCTGTGAATCTGGCAGCGGCAGCTTAGAGCCTGTGGTTG
GSGS ins reverse	CAACCACAGGCTCTAAGCTGCCGCTGCCAGATTCACAGGTGAG
ESLE del forward	GTGGACCTCACCTGTCCTGTGGTTGTGGACCTGAC TCACAATGAC
ESLE del reverse	GGTCCACAACCACAGGACAGGTGAGGTCCACGATTTCATCTCCAACGG
RRNGRR del forward	GAAAGGAGAAGGCCATTGCGCCAAGACCATGCTGACAGCTGTGTGG
RRNGRR del reverse	GCATGGTCTTGGCGCAATGGCCTTCTCCTTTCTTCAACAATCACAAC
SL del forward	CTCACCTGTGAAGAGCCTGTGGTTGTGGACCTGACTCACAATGAC
SL del reverse	CAACCACAGGCTCTTCACAGGTGAGGTCCACGATTTCATCTCCAACGG
RRNGRR to E forward	GGCCAGAGGAAAATGGGGAGGAGTTGCGCCAAGACCATGCTGACAGC
RRNGRR to E reverse	CTTGGCGCAACTCCTCCCCATTTTCCTCTGGCCTTCTCCTTTCTTCA
RR85EEonRR81EE for	GCCAGAGGAAAATGGGGAGGAGTTGCGCCAAGACCATGC
RR85EEonRR81EE rev	ATGGTCTTGGCGCAACTCCTCCCCATTTTCCTCTGGCCT
